# Microglia in neurodegenerative diseases: mechanism and potential therapeutic targets

**DOI:** 10.1038/s41392-023-01588-0

**Published:** 2023-09-22

**Authors:** Chao Gao, Jingwen Jiang, Yuyan Tan, Shengdi Chen

**Affiliations:** 1 Department of Neurology, Ruijin Hospital, Shanghai Jiao Tong University School of Medicine, 200025 Shanghai, China; 2 Lab for Translational Research of Neurodegenerative Diseases, Shanghai Institute for Advanced Immunochemical Studies (SIAIS), Shanghai Tech University, 201210 Shanghai, China

**Keywords:** Neuroimmunology, Neurological disorders, Neurological disorders, Neuroimmunology

## Abstract

Microglia activation is observed in various neurodegenerative diseases. Recent advances in single-cell technologies have revealed that these reactive microglia were with high spatial and temporal heterogeneity. Some identified microglia in specific states correlate with pathological hallmarks and are associated with specific functions. Microglia both exert protective function by phagocytosing and clearing pathological protein aggregates and play detrimental roles due to excessive uptake of protein aggregates, which would lead to microglial phagocytic ability impairment, neuroinflammation, and eventually neurodegeneration. In addition, peripheral immune cells infiltration shapes microglia into a pro-inflammatory phenotype and accelerates disease progression. Microglia also act as a mobile vehicle to propagate protein aggregates. Extracellular vesicles released from microglia and autophagy impairment in microglia all contribute to pathological progression and neurodegeneration. Thus, enhancing microglial phagocytosis, reducing microglial-mediated neuroinflammation, inhibiting microglial exosome synthesis and secretion, and promoting microglial conversion into a protective phenotype are considered to be promising strategies for the therapy of neurodegenerative diseases. Here we comprehensively review the biology of microglia and the roles of microglia in neurodegenerative diseases, including Alzheimer’s disease, Parkinson’s disease, multiple system atrophy, amyotrophic lateral sclerosis, frontotemporal dementia, progressive supranuclear palsy, corticobasal degeneration, dementia with Lewy bodies and Huntington’s disease. We also summarize the possible microglia-targeted interventions and treatments against neurodegenerative diseases with preclinical and clinical evidence in cell experiments, animal studies, and clinical trials.

## Introduction

Microglia, the resident macrophages of the central nervous system (CNS), utilize their specific receptor repertoire to monitor the microenvironment dynamically in the brain.^1^ Microglia phagocytose misfolded proteins, cellular debris, and dying cells to maintain homeostasis.^2^ Additionally, microglia could monitor and protect neuronal functions through microglia-neuron crosstalk.^3^

Microglia have long been considered homogenous cells that respond uniformly to their surroundings. Nevertheless, recent developments in single-cell technologies have revealed multiple microglial states in human and mouse brains related to specific developmental, aging, and disease processes.^4^ For example, single-cell RNA-seq (scRNA-seq) or single-nucleus RNA-seq (snRNA-seq) enabled the identification of microglia clusters by analyzing their transcriptional signatures. Using single-cell mass spectrometry (cytometry by time-of-flight [CyTOF]), more than 40 different surface markers can now be identified at the single-cell level, enabling the characterization of immune cell populations in humans and rodents.^5–7^ These findings indicate that microglia are highly heterogeneous cells that are more complex than previously believed. Both intrinsic factors (species, sex, genetic background, etc.) and extrinsic factors (pathogens, nutrition, microbiota, etc.) influence microglial states.^8^

The terminology “M1” and “M2” microglia is previously widely adopted in microglial research, in which microglia were artificially classified into two opposite types based on findings obtained using in vitro models: the M1 pro-inflammatory and neurotoxic microglia and the M2 anti-inflammatory and neuroprotective microglia.^9,10^ However, this simplistic classification fails to capture the complexity of microglial responses in the context of neurodegenerative disease. Reactive microglia that refer to microglia undergoing morphological, molecular, and functional remodeling in response to brain challenges (i.e., amyloid β [Aβ] or α-synuclein [α-syn] deposition, infected, damaged, or degenerating neurons) have been observed in various neurodegenerative diseases. Nevertheless, previous studies have relied solely on morphological observation or specific immunohistochemical staining markers to detect these reactive microglia, which were found to cluster in close proximity to pathological hallmarks such as amyloid plaques or α-syn deposits in various brain regions of mouse models and human postmortem cases.^11,12^ Recent advances in scRNA-seq and snRNA-seq technologies have identified high spatial and temporal heterogeneity levels and unique disease-related signatures of these reactive microglia without correspondence to the canonical M1/M2 classification in neurodegenerative diseases.^13–15^ For instance, scRNA-seq studies identified a specific microglial response state, called disease-associated microglia (DAMs), in mouse models and human patient specimens of AD.^13,14,16^ Notably, DAMs were localized near Aβ plaques and participated in the clearance of β-amyloid.^13^ Moreover, distinct Aβ and tau-associated microglia signatures have been discovered in AD patients.^15^ These findings suggest that microglia show plasticity when responding to various pathologies, highlighting the need to identify disease-specific microglial states and explore factors influencing them to treat AD and other neurodegenerative disorders effectively.

Overall, with the fast development of techniques, recent research on microglia has remarkably revealed their roles in neurodegenerative diseases. In this comprehensive review, we summarize the research history of microglia, the ontogeny and origin of microglia and their physical functions in the homeostatic brain, highlight the current knowledge of the roles of microglia in neurodegenerative diseases including Alzheimer’s disease (AD), Parkinson’s disease (PD), multiple system atrophy (MSA), amyotrophic lateral sclerosis (ALS), frontotemporal dementia (FTD), progressive supranuclear palsy (PSP), corticobasal degeneration (CBD), dementia with Lewy bodies (DLB), and Huntington’s disease (HD). We also summarize the possible microglia-targeted interventions and treatments against neurodegenerative diseases with preclinical and clinical evidence in cell experiments, animal studies, and clinical trials.

## Research history of microglia

It has been more than 100 years since microglia were first discovered in 1919. In 2019, Sierra et al. wrote a review to mark the 100th anniversary of the discovery of microglia and recounted the milestones in a century of microglia research.^17^ In 1919, Spanish researcher Pı´o del Rı´o-Hortega discovered a new type of glial cell based on his invention of a novel method to stain the brain.^18^ As the tiny size of its soma, so he named it “microglia”. He also found that microglia could phagocytose dendritic spines and cell debris and interact with other cells in the brain parenchyma. Microglia could also proliferate and undergo morphological activation in pathological conditions.^18–22^ In 1939, John Kershman from the Montreal Neurological Institute first analyzed the origin of microglia in the human brain and found that microglia infiltrate from some sites, such as the choroid plexus, during embryonic human development.^23^ In 1968, Georg Kreutzberg’s group discovered the role of microglia in synaptic stripping in pathology.^24^ In 1974, Ibrahim et al. developed a novel method to observe microglia based on histochemical labeling ATPases, which are highly expressed by microglial cells.^25^ In 1986, Dana Giulian and Timothy Baker established the first microglia culture system, which was an important step in manipulating and studying microglial function.^26^ Thereafter, it was found that microglia were involved in pathogenesis by releasing chemokines and cytokines.^27,28^ In 1990, the electrophysiology technique was applied to examine ionic currents in isolated microglia.^29^ In 1992, a BV-2 cell line was established to study microglia in vitro. Although in vitro and ex vivo microglia differ in many functional aspects, the BV-2 cell line is still in use today.^30^ In 1997, by developing microglial-preferring ligands, such as PK11195, “activated” microglia could be directly detected in vivo by positron emission tomography (PET) imaging.^31^ Nonetheless, this method of labeling microglia is not specific. The rationale and limitations of this method will be discussed later in the section “Microglial activation in AD brains”. In 1998, with the development of ionized calcium-binding adaptor molecule 1 (Iba1) antibodies, microglia could be reliably identified in tissue.^32^ Iba1-label has become one of the gold standards for identifying microglia, although it can also be used to label macrophages. In 2005, a heterozygous Cx3cl1^GFP/+^ mouse line was established. Cx3cl1 is selectively expressed in microglia in the brain. Thus, by utilizing this mouse line in conjunction with in vivo imaging techniques, it is possible to directly observe the response of microglia to their surrounding environment.^33,34^

In the past 20 years, microglia research has undergone rapid exponential growth. The advance in technology has contributed significantly to our in-depth understanding of microglia. Here, the progress of our knowledge of microglia identity will be shown as an example. It was in the mid-1970s that the microglia were divided into “resting microglia” and “activated microglia”. The consensus at that time was that microglia remain static under physiological conditions or in the normal brain, showing a ramified phenotype, and these “resting microglia” transform to “activated” under pathological conditions or in the diseased brain, characterized by an ameboid morphological appearance. Nevertheless, in 2005, with the development of a two-photon in vivo imaging system and the establishment of a heterozygous Cx3cl1^GFP/+^ mouse line, researchers found that microglia are not static but rather extraordinarily dynamic and constantly survey the parenchyma with their highly motile processes, even in the absence of pathological challenge. Recently, based on single-cell sequencing and single-cell mass cytometry, studies have identified various microglial states in both normal and diseased brains. Now, microglia are no longer considered to simply switch from ‘resting’ to ‘activated’ in response to injury, disease, or other challenges. Instead, microglia are continuously active, adopt different states and perform different functions in response to the surrounding environment in the context of health or disease.^8,17^ Here are some of the key findings in the last decades: (1) microglial are dynamic and heterogenous; (2) microglia communicate with other cell types in the brain; (3) microglia play both protective and deleterious roles in neurodegenerative diseases; (4) microglia can be reprogramed; (5) peripheral immunity regulate microglial response such as via gut–microbiota–brain axis; and (6) microglia also age.^17^ It is beyond this article’s scope to thoroughly review all progress of microglial research in health and disease, which has been reviewed elsewhere.^35–41^ With the fast development of techniques such as live imaging, single-cell omics, and tools designed to manipulate microglia ex vivo and in vivo, the field is expected to advance rapidly in the coming years.^17^

## Ontogeny of microglia

Microglia were long thought to be of neuroectodermal origin like other glial cells and neurons. Nevertheless, microglia are a unique lineage of tissue macrophages, and it is now well-established that microglia are derived from yolk sac (YS) erythromyeloid precursors (EMPs). These EMPs give rise to YS macrophages, which serve as precursors that inhabit the embryonic brain.^42,43^

In rodents, haematopoiesis contains at least three waves.^41^ There is some overlap in timing and tissues involved in these waves, which could explain why it has always been difficult to determine the ontogeny of microglia and macrophages in the CNS.^44^ The first wave, that is the initial phase of hematopoiesis, termed “primitive” hematopoiesis, begins in the YS blood islands (posterior plate mesoderm) at approximately E7.0 (embryonic day 7.0). Between E7.0 and E8.0, this wave generates primary EMP cells. These primary EMPs in the YS generate YS macrophages, which differentiate into microglia or non-parenchymal macrophages in the CNS and tissue macrophages in the peripheral tissues.^45^ The primary EMPs express the macrophage colony-stimulating factor 1 receptor (CSF1R) and depend on it for survival and differentiation.^42^ The second “transient definitive” wave of hematopoiesis starts in the YS haemogenic endothelium at E8.25, This leads to the emergence of secondary EMPs. Unlike primary EMPs, secondary EMPs lack CSF1R expression but rely on c-myb for their development,^46^ suggesting that secondary EMPs possess distinct molecular properties and/or differentiation potential compared to primary EMPs. The third ‘definitive’ wave of haematopoiesis initiates in the embryo proper at E8.5. This wave generates immature hematopoietic stem cells (HSCs) from the haemogenic endothelium in the para-aortic splanchnopleura region. At E10.5, this region develops into the aorta, gonads, and mesonephros (AGM) region. Fetal HSCs migrate from this region to the liver, where they join the secondary EMPs in producing fetal liver (FL) monocytes.^46,47^ Thus, primary EMPs, secondary EMP-derived fetal liver monocytes, and HSC-derived fetal liver monocytes contribute to all tissue macrophages, with microglia arising solely from primary EMP.^41^

## Origin and development of brain microglia

Microglial cell colonization of the CNS is evolutionarily conserved across vertebrate species and occurs before the formation of the neuroectoderm-derived glial cell types such as oligodendrocytes and astrocytes.^48,49^ EMPs originate in the YS and differentiate into YS macrophages before migrating toward embryonic tissues, including the brain. At E9.5, microglia infiltrate the brain rudiment, entering the leptomeninges and the lateral ventricles to spread throughout the cortex at varying speeds depending on the region and developmental stage.^42,50^ Normal blood circulation is required for YS macrophage seeding of the CNS. Between E8.0 and E10.0, blood vessels form and remodel de novo in the mouse embryo, coinciding with the appearance of YS precursors in the embryo.^51^ Interestingly, sodium-calcium exchanger 1 (NCX1)-deficient mice, which exhibit a defective circulatory system, lack microglial progenitors in the embryonic brain despite with normal YS hematopoiesis. This observation supports the idea that the recruitment of YS progenitors into the brain is mediated by blood circulation.^42^ Yolk sac c-Kit^+^ EMPs developed into CD45^+^c-kit^lo^ CX3CR1^-^ immature (A1) cells and matured into CD45^+^c-kit^-^ CX3CR1^+^(A2) macrophages.^43^ A2 macrophages enter the developing mouse brain via the pial surface at E9.5 and migrate along the abluminal surface through the vasculature to become microglia without a monocyte intermediate.^43,52,53^ Microglial precursors receive instructive signals from the CNS environment once inside the brain parenchyma, which aids in their differentiation.^54^ Amoeboid macrophages eventually become ramified morphology and cover more of the CNS between E14.5 and the first postnatal week.^41^

The microglia within the CNS are maintained by both circulating monocytes and repopulation from CNS-endogenous cells. Despite the decrease in proliferating microglia between E14.5 and E15.5, a significant increase in the total number of microglial cells was observed during this period. This finding indicates the possible existence of an additional source of microglial cells that contributes to the resident microglial population.^54^ Hoxb8 represents a gene of considerable significance in orchestrating the intricate development and functioning of microglia within the brain. At least two progenitor pools for microglia have been demonstrated: canonical non-Hoxb8 microglia and Hoxb8 microglia. Hoxb8 microglia progenitors appear to arise during the second wave of YS hematopoiesis and then enter the AGM region and fetal liver, where their number is greatly increased before they migrate into the developing brain at E12.5. It is estimated that non-Hoxb8 microglia account for 70% of all microglia in the adult brain, significantly outnumbering Hoxb8 microglia, but non-Hoxb8 microglia cannot compensate for the loss of Hoxb8 function in Hoxb8 microglia.^55^ It is of interest to identify the origin of Hoxb8 microglial progenitors, explore their development, migration, infiltration, and functional changes, and further explore their transcriptional profiles and turnover characteristics by proper fate-mapping system, such as tamoxifen-inducing Cre line, in combination with scRNA-seq and use of reporter cell lines, etc. Human amoeboid microglia infiltrate the developing cerebral cortex through multiple routes, including the pial surface, ventricles, and choroid plexus at 4.5 gestational weeks (gw). These microglia exhibit both radial and tangential migration, directing themselves toward the immature white matter, subplate layer, and cortical plate.^48,56^ At 12-13 gw, a second wave of microglial invasion via the vasculature is limited to the white matter.^48^ Evidence also showed repopulation of microglia from CNS-endogenous cells following global microglia depletion, which contributes to the dynamic regulation of the microglia population in the adult mouse brain.^57,58^ Studies have revealed that in mice, microglia undergo proliferating (Iba1^+^ BrdU^+^) at a rate of 0.69% after a single pulse of BrdU (per average of about 96 days), whereas in humans, this rate is ~2%.^59^ The turnover rate of microglia differs in various regions of the brain in mice, with the olfactory bulb, hippocampus, and cortex in mice undergoing complete renewal in 8, 15, and 41 months, respectively.^60^ Resident microglia in adults are known to maintain their cell density by balancing proliferation and apoptosis. In humans, the average lifespan of cortical microglia is ~4.2 years.^61^ Microglial self-renewal appears stochastic, with no regional hot spots, but this process switches to clonal proliferation during pathology.^60^

## Microglia in the homeostatic brain

### Factors for microglia development and maturation

Multiple factors regulate the development and maturation of microglia. PU.1, a member of the ETS family, and interferon regulatory factor (IRF8) both function as heterodimers in determining the phenotype of brain macrophages and are essential for the early development of YS microglia precursors.^43,62,63^ Runx1, expressed in a subset of microglia during early postnatal forebrain development, regulates myeloid cell proliferation and differentiation.^64^ Runx1 directly binds to the upstream regulatory region of the PU.1 gene, regulating its expression during embryonic and adult hematopoiesis.^65^ The colony-stimulating factor 1 receptor (CSF1R) is another key regulator for microglia development and maintenance.^66^ Mice lacking Csf1R exhibit impaired brain architecture and microglia-depleted embryos.^67^ IL-34, a tissue-restricted ligand of CSF1R, is also required for the development of microglia.^68^ Mature microglia also require CSF1R signaling, as demonstrated by the significant loss of microglia in adult mice treated with Csf-1R inhibitors.^69^ CSF1R ligands are major components in all protocols for generating induced pluripotent stem cell (iPSC)-derived microglia, underscoring that CSF1R signaling also plays a significant role in microglia fate specification.^70^ Transforming growth factor-β (TGF-β) has been proposed as a critical brain-derived signal for microglial specification. When primary microglia are cultured with CSF1 and TGFβ, a significant increase in the expression of microglial signature genes is observed compared to CSF1 alone.^71^

### Microglial expansion in CNS pathologies

Microgliosis refers to the reactive proliferation of microglial cells in response to pathological conditions. To recover from injury or damage, clones of microglia are reorganized by microglial cell migration and cell death. For example, in response to clinical recovery after facial nerve axotomy, certain microglia near the lesion in the facial nucleus underwent apoptosis or were eliminated through cell migration during the re-establishment of microglial steady state.^60^ scRNA-seq after facial nerve axotomy in mice revealed the genes that were related to immune response, neuronal cell death, and microglia migration were upregulated, whereas the genes associated with the homeostatic microglial signature, such as Cst3, demonstrate downregulation.^72^ Although the specific mechanisms underlying the migration and cell death of excess microglia due to clonal expansion remain unclear, these observations indicate that microglia tend to reorganize and restore their homeostasis during clinical recovery.

Recently, microglia and their blood-borne counterparts have been identified as crucial players in disease-associated brain microenvironments and have been implicated in neurodegenerative disease progression.^73^ The infiltration of monocyte-derived macrophages (MDMs), which have a higher phagocytic activity than microglia, promotes tissue repair and the resolution of inflammation.^74^ Various methods can be employed to distinguish resident microglia from infiltrated monocytes. In a study, researchers employed a CyTOF panel consisting of 57 markers to characterize the human CNS-resident microglia (huMG) in various brain regions, peripheral blood mononuclear cells (PBMCs), and immune cells from cerebrospinal fluid obtained postmortem from nine donors. Their analysis revealed a distinctive signature specific to huMG, enabling differentiation from mononuclear cells. Notably, CD44 expression was exclusively observed on infiltrating cells rather than resident myeloid cells. The study also detected three subpopulations of microglia that vary regionally and can be distinguished by different levels of specific markers. One subpopulation consisted of microglia increased expression of proteins associated with proliferation (cyclin, cyclin B, Ki-67) and was predominantly found in the subventricular zone (SVZ) and thalamus. The other two microglial clusters originated from the frontal and temporal lobes, respectively. Both clusters exhibited upregulated CD206 but they differed in the levels of CD64 and EMR1.^7^ Another approach involved using CD11b^+^CD45^high^ and CD11b^+^CD45^low^ as markers for peripheral monocytes/macrophages and microglia, respectively^75^ and found that during the early stages after focal transient ischemia, microglia exhibit a highly branched morphology and show a faint staining intensity (CD45low). In contrast, infiltrated leukocytes display a round-shaped morphology and exhibit a strong, well-contrasted staining (CD45high). These distinct characteristics allow for differentiation between microglia and infiltrated leukocytes.^76^ It is worthy to further explore different states and different functions of resident microglia and periphery-derived microglia-like cells in the CNS in neurodegenerative disease.

### Functions of microglia during homeostasis

Microglia in a homeostatic state use their ramified processes to survey the microenvironment in real time for potential signals that warrant further action. Mature microglia in the postnatal brain use a wide range of surface molecules to respond quickly to their extracellular environment, including cytokines, chemokines, purines, hormones, and neurotransmitters.^77^ Similar to other macrophages residing in tissues, microglia express common markers such as the fractalkine receptor CX3CR1, CSF1R, the integrin CD11b, surface glycoproteins F4/80 and CD68, ionized calcium-binding adaptor molecule 1 (Iba1), and pan-hematopoietic CD45. However, the expression levels of these markers are generally lower than those observed in perivascular macrophages and blood monocytes at steady state.^78^ Microglial activation is tightly regulated through receptor-ligand interactions, such as CX3CR1-CX3CL1 and SIRPa-CD47.^79^ Additionally, in the adult brain, microglia display remarkable efficiency in clearing dead cells and excess cellular material, and microglial phagocytosis shapes adult hippocampal neurogenesis.^80^ TAM receptor tyrosine kinases Mer and Axl and their ligands Gas6 and protein S regulate the process of microglial phagocytosis. In adult mice, the absence of microglial expression of Axl and Mer leads to a marked accumulation of apoptotic cells, specifically in neurogenic regions of the CNS.^81^

A rising number of investigations have shown microglial roles in synapse formation, pruning and elimination, and regulation of synaptic function. Synapse elimination occurs during normal brain development, which involves the removal of unnecessary excitatory and inhibitory synaptic connections.^82^ This elimination process is vital for the formation of mature and efficient neuronal circuits during normal brain development.^83^ The traditional complement cascade proteins C1q and C3, broadly expressed in the developing brain, localize to specific subsets of immature synapses and mediate their elimination.^84^ Microglia can phagocytose complement-tagged synapses through the C3-C3 receptor(C3R) pathway, which is crucial for accurate synaptic connection. Importantly, interruption of this pruning mechanism causes long-lasting damage to brain circuitry and synaptic connections.^84^ Recent research suggests that microglia may respond to astrocyte-derived interleukin-33 (IL-33) to promote synaptic pruning in regions such as the hippocampus and the reticular thalamic nucleus. Knockout mice lacking IL-33 revealed impairments in synaptic elimination during development, suggesting the contribution of astrocytes in regulating microglial-mediated synaptic pruning.^85^ Microglia also play a crucial role in the modulation of synaptic plasticity. Microglia could enhance synaptic plasticity through the expression and release of brain-derived neurotrophic factor (BDNF) via the microglial phosphatidylinositol 3-kinase (PI3K)/BDNF signaling pathway. BDNF, as a downstream effector of microglial PI3K, increases the plasticity of dendritic spines in the adult cortex.^86,87^ Microglia can also secrete other neurotrophic factors and cytokines to regulate synaptic plasticity, such as TNFα^.^^88^ Additionally, DAP12 signaling participates in the microglia-mediated regulation of synaptic plasticity. DAP12 is exclusively expressed in microglia in the murine brain, and DAP12 deficiency results in a marked impairment of synaptic plasticity.^89^

Dysregulation of synaptic elimination is involved in the pathogenesis of neurodegenerative diseases.^90^ Synaptic loss precedes neuronal loss and is considered a more accurate indicator of cognitive decline in AD.^91^ In neurodegenerative diseases, reactive microglia found near protein aggregates such as Aβ plaques are involved in synapse loss and neuronal damage. Eliminating microglia or attenuating microglial activation in neurodegenerative diseases restored spine number and synaptic integrity and improved functional outcomes.^92^ In AD brains, microglia mediate aberrant synapse loss via complement mediators (especially C1q and C3),^93,94^ as well as through the triggering receptor expressed on myeloid cells 2 (TREM2) signaling.^95^ Additionally, microglia phagocytosis of synapses is also affected by astrocytes. Selective removal of astrocytic APOE4 decreased microglial phagocytosis of synaptic elements in the tau transgenic mouse model.^96^ Thus, microglia play an indispensable role in regulating the formation, plasticity, and elimination of synapses throughout development and adulthood. Importantly, microglia dysfunction can be an active inducer of the initiation and progression of various neurodegenerative diseases.

### Microglia-neuron crosstalk

Microglia communicate with nearly all cell types in the brain to facilitate developmental process, maintain homeostasis, assist in tissue repair, and contribute to the pathogenesis of diseases.^53,97^ Reactive microglia undergo proliferation and accumulate in regions with high densities of apoptotic neurons as phagocytes to promote neuronal turnover during developmental cell death and mediate the regulation of synaptic function.^98^

Microglia maintain neuronal survival and regulate neurogenesis throughout both the prenatal and postnatal stages of development. Microglia limit the production of cortical neurons by phagocytosing neural precursor cells^99^; at the same time, microglia also promote neurogenesis, as microglia depletion in mice reduces basal progenitors into the cerebral cortex.^100^ Microglia-derived insulin-like growth factor-1 (IGF1) maintained neuronal survival.^101^ Microglia can also prevent neuronal hyperexcitability as genetically inhibition of Gi in microglia increases hypersynchrony upon physiologically evoked neuronal activity.^102^ Microglia-derived IL-1β enhances presynaptic glutamate release by promoting the NMDAR-dependent synthesis of arachidonic acid and prostaglandins.^103^ Neuronal CD200 interacts with CD200 receptor (CD200R) expressed on microglia and modulates microglial activation.^104^ Meanwhile, the CD200/CD200R signaling pathway also contributes to the regulation of synaptic plasticity.^105^ Neuron also induces microglial process extension, and the mechanism involves the neuronal NMDA receptors activation which causes neuronal ATP release, and P2Y12 receptors mediated microglial response.^106,107^

### Microglia-astrocyte crosstalk

The interaction between reactive microglia and astrocytes is critical in the development of neuroinflammation. Although the canonical M1/M2 (microglia) and A1/A2 (astrocyte) classifications are not accurate in describing the states of microglia and astrocytes, this classification is helpful to elucidate the interaction of microglia and astrocytes and will be adopted here. Microglia and astrocytes exhibit two polarization states: pro-inflammatory (M1 and A1) and anti-inflammatory (M2 and A2). Microglia are more susceptible to pathogens or damage such as LPS or stroke. Activation of pattern recognition receptors (PRRs) via pathogen-associated molecular patterns (PAMPs) or damage-associated molecular patterns (DAMPs) triggers microglia M1 phenotype.^108^ Microglia display a diverse set of toll-like receptors (TLRs), whereas astrocytes primarily express TLR3, with minimal expression of TLR1, TLR4, TLR5, and TLR9 and no expression of TLR2, TLR6, TLR7, TLR8, and TLR10.^109^ The relatively low expression of TLRs in astrocytes suggests they may have limited ability to respond directly to various pathogens. Instead, they rely on microglia to detect pathogens and communicate with astrocytes to induce their activation. Specifically, in the case of TLR4 activation triggered by LPS, microglia are directly involved in initiating or facilitating astrocytic responses by releasing mediators. This highlights the crucial role of microglia-astrocyte crosstalk in the CNS’s response to insults, injuries, or inflammatory stimuli.^110^ Microglia have the potential to enhance the inflammatory activation of astrocytes by increasing the expression level of cytokines and chemokines, specifically through the activation of nuclear factor-κB (NF-κB) signaling.^111^ Reactive microglia produce IL-1α, TNFα, and C1q, which induce the neurotoxic A1 astrocytes phenotype conversion.^112^ Once A1 astrocytes are induced, they lose their essential functions such as supporting neuronal survival, and they also promote neuroinflammation, which contributes to the progression of neurodegenerative diseases.^113^ Reactive M2-like microglia produce the anti-inflammatory cytokine IL-10, which binds to the IL-10 receptor (IL-10R) mainly expressed in A2 astrocytes. This interaction enables astrocytes to release TGF-β, reducing microglial activation.^114^ Communication via extracellular vesicles (EVs) has recently been identified as a critical pathway for CNS cells because EVs may be released and taken up by various cell types. EVs are essential mediators of microglia-astrocyte interaction. Astrocyte-derived ATP induces the formation and the shedding of EVs and IL-1β release in nearby microglia, triggering a neuroinflammatory response.^115^

### Microglia-oligodendrocyte crosstalk

Oligodendrocyte precursor cells (OPCs), highly proliferative cells that mature in separate waves, give rise to myelinating oligodendrocytes. Microglia play an essential role in the proper development and homeostasis of OPCs and oligodendrocytes.^116^ Reactive microglia of the SVZ release TNFα, IL-1β, IL-6, and IFN-γ, promoting oligodendrocyte development. In contrast, a reduction in the levels of these cytokines impairs oligodendrogenesis.^117^ A CD11c^+^ microglial subset was identified in the developing brain that predominates in the primary myelinating areas. These CD11c^+^ microglia express genes for neuronal and glial survival, migration, and differentiation. These CD11c^+^ microglia, characterized by their amoebic shapes along white matter pathways such as the corpus callosum and cerebellum, serve as a primary source of IGF1 and other factors involved in neurogenesis and myelinogenesis, which exert their effects on oligodendrocytes in the newborn brain.^118^ Thus, the interaction between early postnatal microglia and OPCs/oligodendrocytes in the white matter at a specific developmental stage is critical in supporting proper myelin synthesis. Besides, fractalkine-dependent microglial pruning of OPCs is indispensable for proper myelination. Mice lacking fractalkine receptor show a reduction in microglial phagocytosis of OPCs, and increased numbers of oligodendrocytes but reduced myelin thickness.^119^ Additionally, in the microglia-oligodendrocyte cocultures system, microglia stimulate oligodendrocytes to synthesize sulfatide, a myelin-specific galactolipid, along with myelin proteins myelin basic protein (MBP) and proteolipid protein (PLP).^120^

## Microglial dysfunction in neurodegenerative diseases

In the CNS, microglia, as the first line of immune defense, constantly survey their environment and interact with neurons, astrocytes, oligodendrocytes, and infiltrating immune cells. In the homeostatic brain, microglia exert roles in synapse pruning, injury repair, homeostasis maintenance, phagocytosis, support of other glial cells, and communication with other cells. Microglia respond to CNS injuries and diseases with complex reactions, commonly called “activation.” Microglia activation was observed in various neurodegenerative diseases. In the early years of microglial research, microglial activation was detected by morphological observation as they transformed from their ramified phenotype in the normal brain to ameboid morphological appearance in the diseased brain. However, microglia activation is more varied and dynamic than ever anticipated, both in -omics features and functional consequences, indicating that microglia respond differently in different diseases. In the last section, we reviewed microglial functions in the homeostatic brain to provide context for microglial changes in neurodegenerative diseases. In this section, we present the diversity of microglia states responding to pathological conditions and highlight the current knowledge of the roles of microglia in neurodegenerative diseases. Meanwhile, we also summarize some therapeutic approaches for neurodegenerative diseases that target microglia.

### Alzheimer’s disease

Alzheimer’s disease (AD) is the most common neurodegenerative cause of senile dementia, accounting for 60–70% of dementia cases. In 2019, there were more than 55 million dementia patients worldwide, and the number is expected to rise to 139 million by 2050, according to the World Alzheimer Report 2022.^121^ The primary pathological features of AD are extraneuronal amyloid plaques formed by the deposition of Aβ peptide in the brain and intracellular neurofibrillary tangles (NFTs) caused by abnormal aggregation of tau protein.^122^ Mechanismly, various factors, including genetics, amyloid protein, tau, ApoE, and neuroimmune activation, are involved in the pathogenesis of AD.^123^ Recently, genome-wide association studies (GWAS) have demonstrated that most AD risk genes are highly or exclusively in microglia,^124^ suggesting that microglia play an essential role in AD development.

#### Microglial activation in AD brains

Several studies conducted on autopsy tissues of AD patients and controls showed that microglial activation was observed in AD brains,^125,126^ and microglial activation was significantly higher within Aβ plaques compared with plaque-free cortical areas.^125,127^ Microglial activation has been detected in vivo by PET imaging. Transporter protein (TSPO) is an 18 kDa translocator protein, expressed at low levels on the outer membrane of mitochondria in the glial cells under physiological conditions. However, during neuroinflammation, the expression level of TSPO is significantly upregulated in these reactive glial cells, especially in microglia. Therefore, numerous studies have utilized radiotracers that specifically bind to TSPO to visualize the reactive microglia directly.^128,11^[C](R)-PK11195 (PK), as the first-generation TSPO PET tracer, has been extensively used to study microglial activation. However, several factors have limited its widespread use. The first challenge is related to the low signal-to-noise ratio, which makes it difficult to detect subtle changes in neuroinflammation. Several sites in the blood, including plasma proteins, monocytes, and platelets, bind to 11[C](R)-PK11195, resulting in a low brain permeability and, therefore, a low signal-to-noise ratio.^129^ Secondly, TSPO is also expressed by other cell types, such as astrocytes and vascular endothelial cells in the brain, indicating its non-specificity. Cellular sources and subcellular localization of TSPO expression in healthy and diseased brain has been reviewed in previous studies.^130,131^ Thirdly, the relatively short half-life of carbon-11 also limits the clinical usefulness of^11^[C](R)-PK11195.^132^ Over the past few years, second and third-generation TSPO tracers have been developed to address these limitations. Unfortunately, these new radioligands have not been without their problems. The first factor concerning the rs6971 polymorphism of human TSPO polymorphisms affects the binding affinities of radiotracer.^133^ Furthermore, the problem of non-specific binding remains. Importantly, TSPO is more suitable as a biomarker of neuroinflammation than a marker of microglial activation.^131^ However, many studies have used TSPO-PET imaging to detect microglial activation in a diverse range of neurodegenerative diseases.^132^ Consistent evidence using PET imaging indicated microglial activation in the brains of AD patients. Using [^11^C](R)-PK11195, [^11^C]-PBR28, or some other TSPO-specific radiotracers, AD patients all showed significantly increased regional TSPO ligand binding in the cortices compared to controls.^134–136^ In addition, microglial activation was found in the white matter.^137^ Microglial activation has also been detected by PET in patients with mild cognitive impairment (MCI).^138–140^ In MCI, microglial activation was positively correlated with amyloid load.^141,142^

#### Microglia response to AD pathology

Previous studies detected reactive microglia by morphological observation and specific immunohistochemical staining markers. These reactive microglia were found to be clustered near Aβ plaques in various brain regions of AD mice and human postmortems.^11,12^ The in vivo imaging study also found that microglial activation correlated with tau and amyloid in AD.^143^ In recent years, with the development of scRNA-seq and snRNAseq technologies, microglia in various states were identified, significantly advancing our knowledge of microglia responses to pathological hallmarks in AD. In an AD mouse model (5×FAD transgenic mice which recapitulate major features of AD amyloid pathology), Keren-Shaul et al.^13^ first identified a subgroup of microglia in AD, termed disease-associated microglia (DAM), which participated in the clearance of Aβ. DAMs were localized near Aβ plaques, which has also been validated in AD postmortem brain samples. In AD patients, snRNA-seq of the occipital cortex and the occipitotemporal cortex from AD patients and controls identified three clusters: homeostatic microglia, AD1-microglia, and AD2-microglia. The occipital cortex contained Aβ pathology, with no or low-level tau pathology, while the occipitotemporal cortex contained both Aβ pathology and tau pathology. AD1-microglia were localized near Aβ plaques and were strongly correlated with the tissue Aβ load. Gene ontology analysis indicated that AD1-microglia showed similarities with DAM signatures in the 5×FAD mouse model. Both were associated with “phagocytosis”, “lipid localization”, and “cell migration. AD2-microglia possibly have neurotrophic functions.^15^ Besides, Nguyen et al. characterized microglia in various states in AD brains: homeostatic microglia, amyloid responsive microglia, dystrophic microglia, and motile microglia, among which the amyloid responsive microglia relied on triggering receptor expressed on myeloid cells-2 (TREM2) and APOE signaling.^144^ Another study used snRNA-seq to comprehensively characterize transcriptomes in microglia nuclei isolated from neuropathologically defined AD and control brains with a range of Aβ and phosphorylated (p)-Tau pathology. The study found that microglial transcripts were most highly positively associated with tissue Aβ and tissue pTau density.^145^ These results suggest that microglia respond to the pathology of AD. In the early stage of the disease, this response may be protective. The reactive microglia migrate to the vicinity of pathological deposits such as Aβ or tau and then engulf and eliminate them. However, when these growing pathological deposits chronically stimulate microglia, the protective microglia may convert to dysfunctional microglia, aggravating the disease progression of AD.

#### Microglial roles in the pathogenesis of AD

##### Effect of microglia on Aβ pathology

Considerable evidence has found that microglia promote the uptake and degradation of Aβ. For example, LC3-associated endocytosis (LANDO) in microglia facilitates Aβ receptor recycling, increasing Aβ surface receptors, thus promoting Aβ clearance, and in contrast, LANDO-deficient AD mice induced neurodegeneration and memory deficits.^146^ With aging, the Nogo receptor (NgR) expression on microglia increased, impairing microglial phagocytosis and clearance of Aβ. In contrast, NgR-deficient AD mice reduced amyloid burden and improved cognitive impairment.^147^ BACE-1 inhibition in microglia facilitated the microglia phenotype transition from homeostatic to stage 1 disease-associated microglia (DAM-1) signature^148^ and thus enhanced amyloid clearance and improved cognitive performance in AD mice.^149^ In addition, microglia interact with astrocytes to promote Aβ clearance. After recognizing Aβ deposits, microglia increased their expression of IL-3Rα, the specific receptor for IL-3. Astrocyte-derived IL-3 bound to the upregulated IL-3Rα in microglia, enhancing microglial migration toward Aβ deposits and the Aβ aggregates clearance.^150^ APOE isoforms also affect the phagocytosis of Aβ by microglia. Compared with APOE4, APOE3 lipoproteins induce faster microglial migration towards Aβ, facilitate Aβ uptake, and ameliorate cognition.^151^

**Fig. 1:**
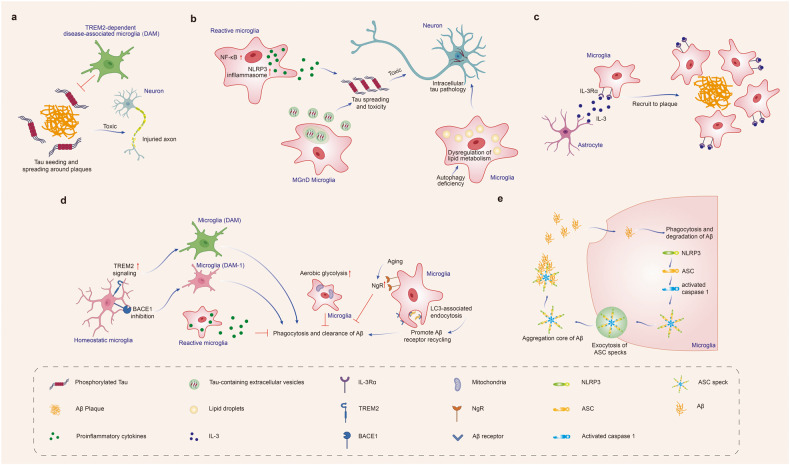
Effect of microglia on Aβ and tau pathology in Alzheimer’s disease. Microglia phagocytose Aβ and tau, limit propagation of Aβ and tau pathology. Under pathological conditions, microglia could also accelerate Aβ and tau spreading and lead to neurodegeneration. **a** TREM2-dependent DAM limits tau seeding and spreading around plaques. **b** Reactive microglia drive tau spreading and toxicity by promoting neuroinflammation, such as activating NLRP3 inflammasome or inducing NF-_k_B signaling. Microglial autophagy deficiency leads to dysregulation of lipid metabolism, thus increasing intraneuronal tau pathology and its spreading. MGnD microglia, which is common in neurodegeneration, hypersecrete EVs containing pTau, accelerates tau propagation. **c** Microglia increase their expression of IL-3Rα after recognition of Aβ deposits. Astrocyte-derived IL-3 binds to the upregulated IL-3Rα in microglia, enhancing microglial migration toward Aβ deposits, and the clearance of Aβ aggregates. **d** TREM2 promotes the conversion of microglia to the DAM phenotype, and BACE-1 inhibition in microglia facilitates the microglia phenotype transition from homeostatic to DAM-1 signature. DAM and DAM-1 phenotypes enhance amyloid clearance. LC3-associated endocytosis (LANDO) in microglia facilitates Aβ receptor recycling, increases Aβ surface receptors, and thus promotes Aβ clearance. In contrast, the microglia with enhanced aerobic glycolysis, and NgR expression on microglia increased with aging inhibit the phagocytosis and clearance of Aβ. **e** Microglia facilitate Aβ spreading. Aβ induces immune system activation and the formation and release of ASC specks. After being released from microglia, ASC specks bind to and promote the cross-seeding of Aβ, leading to amyloid seeding and spreading. Created with https://BioRender.com

Although the above studies have shown that microglia could phagocytize Aβ and reduce amyloid plaque deposition and neurodegeneration, some studies have also found that the phagocytosis of Aβ by microglia promoted plaque development.^152^ In AD mice, sustained microglial depletion with CSF1R inhibitor reduced plaque development.^32^ Besides, microglia facilitate Aβ spreading. Aβ activates the immune system and induces the formation and release of apoptosis-associated speck-like protein containing a caspase activation and recruitment domain (CARD) (ASC) specks. After being released from microglia, ASC specks bind to and promote the cross-seeding of Aβ, leading to amyloid seeding and spreading of amyloid pathology.^153^ To examine whether microglia contribute to Aβ propagation, d’Errico P et al. using transplantation of wild-type (WT) neurons, found that Aβ entered WT grafts accompanied by microglia infiltration and in vivo imaging revealed that microglia were carries of Aβ pathology in previously unaffected tissue^154^ (Fig. 1).

##### Effect of microglia on tau pathology

Microglia limit Aβ-associated tau seeding and spreading in AD mouse models. TREM2 has been reported to promote the conversion of microglia to the DAM phenotype, which is responsible for Aβ phagocytosis.^13^ Similarly, with the presence of Aβ, TREM2-dependent activation of the DAM phenotype can also limit tau pathology propagation. On the other hand, microglia could also drive tau spreading and toxicity by promoting neuroinflammation, such as activating NACHT-, LRR- and pyrin (PYD)-domain-containing protein 3 (NLRP3) inflammasome^155^ or inducing NF-kB signaling.^156^ In addition, autophagy is defective in AD microglia.^157^ Microglial autophagy deficiency can lead to dysregulation of lipid metabolism, induce microglia into a pro-inflammatory state, and as a result, enhance intraneuronal tau pathology and its spreading.^158^ The above evidence suggests that microglia-mediated neuroinflammation is detrimental in accelerating tau pathology. Besides, it has been suggested that EVs as potential carriers propagate misfolded proteins, such as tau and Aβ in AD and α-syn in PD.^159^ In a humanized APP mouse model, MGnD microglia, a class of disease-reactive microglia common in neurodegeneration, hypersecrete EVs containing phosphorylated tau (pTau), accelerating tau propagation.^160^ In contrast, inhibiting microglia secretion of tau-containing EVs alleviated tau pathology and cognitive impairment in P301S tau transgenic mice^161^ (Fig. 1).

##### Impact of TREM2 on microglia responses to AD pathology

*TREM2* is expressed highly and exclusively in microglia in the brain.^162^ GWAS showed that the R47H variant of *TREM2* was associated with a 2- to 4-fold increased risk for the development of AD.^163,164^ Several other *TREM2* variants that affect the expression of TREM2 also increased the risk of AD, including R62H, T66M, H157Y, and D87N.^165–168^ As a result of the genetic association of *TREM2* variants with AD, how TREM2 impacts the microglial response to AD pathology has been studied.

TREM2-dependent microglial activation is critical to sustaining microglia defense against Aβ and tau pathology. Loss of TREM2 function impaired Aβ phagocytosis by microglia and increased amyloid seeding in AD mouse models.^169,170^ Conversely, enhancing TREM2 signaling by TREM2 agonist antibody, direct delivery of the TREM2 gene, or stimulating other pathways to increase TREM2 expression in the brains of AD mice enhanced Aβ phagocytosis and improved cognitive behaviors.^171–173^ As for the underlying mechanism, Aβ binds to microglial TREM2, which activates TREM2 signaling and lead to the enhanced phagocytosis of microglia.^174^ Additionally, sc-RNAseq revealed that TREM2 promoted the conversion of microglia to the DAM phenotype, which is responsible for Aβ phagocytosis.^13^ Although studies have found a protective role for TREM2 in response to amyloid pathology, the opposing roles for TREM2 have been reported in mouse models of tauopathies. The evidence supporting the protective role is that *TREM2* knockout (KO) or *TREM2* R47H variant dramatically enhanced tau seeding and spreading around plaques in AD mice.^175–177^ But other studies found that TREM2 deficiency significantly reduced brain atrophy and prevented microglial activation in tau transgenic mice.^178,179^ Notably, the impact of TREM2 on Aβ and tau pathology may vary at different disease stages. APP/PS1 mice treated with *Trem2* knockdown antisense oligonucleotides (ASOs) through the ventricles at late stages exhibited a 50% reduction in plaque load. In contrast, administration of ASOs at early stages did not affect plaque load.^180^ Responding to tau pathology, in the early stages of AD, TREM2 may suppress tau seeding, but later in AD, it may aggravate tau propagation.^178,179,181,182^

TREM2, a single-pass transmembrane receptor, undergoes proteolytic processing and the soluble variant of TREM2 (sTREM2) is released from the cell via shedding by ADAM protease following proteolytic processing.^183^ sTREM2 can be detected in human plasma and CSF,^184–186^ and clinical evidence showed that sTREM2 is becoming a valuable marker of AD pathology and cognitive decline. High CSF sTREM2 was associated with slower rates of Aβ accumulation,^187^ and higher CSF sTREM2/p-Tau was associated with slower cognitive decline,^188^ which supports the hypothesis that microglia and sTREM2 play a protective role in AD. sTREM2 is thought to be protective by (i) stimulating microglial recruitment, activation, and phagocytosis of Aβ (ii) inhibiting secondary nucleation of Aβ fibrillization and preventing neurotoxicity, (iii) binding of sTREM2 to fibrils to enhance microglial uptake of fibrillar Aβ.^189–191^ However, in opposition to the protective role of sTREM2 in AD, the mutation p.H157Y located at the cleavage site of TREM2 extracellular domain significantly increased TREM2 shedding with elevated sTREM2 levels in the brain and serum but associated with increased AD risk.^192,193^ Additionally, experimental evidence revealed that sTREM2 directly bound to neurons in mouse models of AD^194^ and inhibited LTP induction.^195^ Together, these novel insights into the function of sTREM2 are important to deepen our understanding of the complex biology of TREM2 and microglia in AD.

Overall, microglia are a double-edged sword in AD. Microglia phagocytose Aβ and tau, limit propagation of Aβ and tau pathology, and can also accelerate Aβ and tau spreading and lead to neurodegeneration. Future research will focus on precisely regulating microglia and promoting their conversion into a protective phenotype.

##### Dysfunctional microglia impair neuronal activity

Microglia have surveillance functions that closely interact with neurons to regulate their activity. Disruption of this network can lead to neurodegeneration. The E4 allele of APOE increases the risk of developing late-onset AD.^196^ Microglia harboring an APOE4 allele showed altered cellular metabolism, increasing intracellular and extracellular lipid accumulation. The extracellular lipid droplets directly decreased neuronal activity by increasing inward K^+^ currents.^197^ Microglia could also lead to synaptic dysfunction in vivo and in vitro by releasing Aβ-containing EVs.^198^

Neuronal activity depends on the precise regulation of synapse formation and elimination. However, in AD brains, microglia dysregulation contributes to spine loss. Cerebrovascular damage is one of the key features of AD.^199^ Fibrinogen leaked from the site of cerebrovascular damage into the brain and subsequently bound to the receptor CD11b on the surface of microglia. The interaction of fibrinogen and CD11b mediated microglial activation led to spine loss and promoted cognitive deficits in the 5×FAD mice. Genetic elimination of the fibrinogen binding motif to CD11b ameliorated the above pathological processes and cognitive impairments.^200^ Microglia phagocytosis of synapses is also affected by astrocytes. Selective removal of astrocytic APOE4 decreased microglial phagocytosis of synaptic elements in the tau transgenic mouse model.^96^

**Fig. 2:**
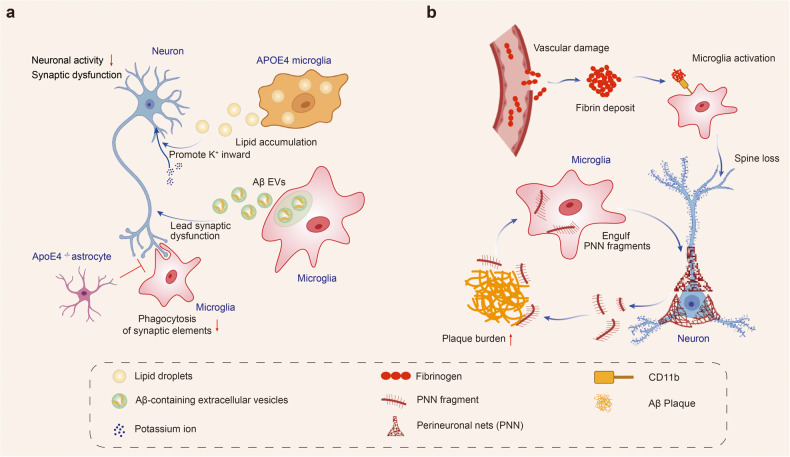
Dysfunctional microglia impair neuronal activity in Alzheimer’s disease. Dysfunctional microglia in AD promotes the clearance of synapses and PNNs and impairs neuronal plasticity and activity. **a** Microglia harboring an APOE4 allele shows altered cellular metabolism with increased intracellular and extracellular lipid accumulation. The extracellular lipid droplets directly decreased neuronal activity by increasing inward K+ currents. Microglia release Aβ-containing EVs and lead to synaptic dysfunction. Microglial phagocytosis of synapses is affected by astrocytes. Selective removal of astrocytic APOE4 decreases microglial phagocytosis of synaptic elements. **b** Fibrinogen leaks from the site of cerebrovascular damage into the brain and subsequently binds to the microglial surface receptor CD11b. The interaction of fibrinogen and CD11b mediates microglial activation and leads to spine loss. Microglia engulf perineuronal nets (PNN) and promote plaque-dependent PNN loss. Created with https://BioRender.com

In addition to eliminating neuronal synapses, the removal of perineuronal nets (PNNs) by microglia is also involved in the pathogenesis of AD. PNNs, components of the extracellular matrix (ECM) surrounding the soma and dendrites of various neuronal cell types in mammals, play important roles in controlling plasticity in the CNS. Removal of PNNs affects functional recovery after CNS injury.^201^ In the 5×FAD mouse model and human cortical tissue, extensive loss of PNNs has been observed, and the loss of PNNs was proportional to plaque burden. Chronically depleting microglia before and during plaque development in two AD transgenic mouse models significantly reduced PNN loss, indicating that microglia promote plaque-dependent PNN loss.^202^ The above studies indicated that dysfunctional microglia in AD promoted the clearance of synapses and PNNs and impaired neuronal plasticity and activity (Fig. 2).

### Parkinson’s disease

Parkinson’s disease (PD) is the second most common neurodegenerative disease after AD, characterized by motor symptoms consisting of bradykinesia, resting tremor, rigidity, and postural instability and non-motor symptoms including hyposmia, constipation, sleep disorders, and depression. Prevalence and incidence rates of PD in Europe are estimated at 108–257/100,000 and 11–19/100,000, respectively.^203^ The number of patients with PD in China is estimated to be 3.62 million.^204^ Pathologically, PD is characterized by the loss of dopaminergic neurons in the substantia nigra pars compacta (SNpc) as well as the accumulation of misfolded α-syn in Lewy bodies. α-syn initially exists as a non-toxic and soluble monomer state. However, its aggregation results in a gain of toxic function.^205^ α-syn fibrils are the main form of α-syn in Lewy bodies. Several genetic and environmental factors regulate the conversion of α-syn monomers to α-syn fibrils, which involves various cellular and biochemical events.^206^ The cause of PD is unknown in most cases, but genetic mutations in the PARK genes and environmental factors such as pollutants, pesticides, heavy metals, and infections may increase the risk.^207^ Two types of PD models are currently used in research: neurotoxin-induced and transgenic. Neurotoxin-induced models use chemicals such as rotenone, 6-hydroxydopamine (6-OHDA), 1-methyl-4-phenyl-1,2,3,6-tetrahydropyridine (MPTP), and paraquat to induce PD-like symptoms. Transgenic models involve genetic modification of PD-related genes such as SNCA, LRRK2, PINK1, PRKN, and DJ-1.^208^

#### Microglia response to PD pathology

Microglial activation begins early and persists throughout the course of PD.^209^ In 1988, reactive microglia were first observed in the substantia nigra (SN) of postmortem brain tissue from PD patients.^210^ Other microglial activation markers, such as pro-inflammatory enzymes like iNOS and COX^211,212^ and phagocytosis-associated marker CD68,^213^ were also upregulated in PD patients. Additionally, PET scans of PD patients revealed widespread microglial activation.^214^ Notably, microglial activation was discovered not only in individuals with long-term illnesses but also in patients who were just diagnosed.^214^

Microglia in the SN of PD patients showed a higher proportion of amoeboid morphology, which is indicative of a reactive state.^215^ It was previously considered that microglia became reactive either towards the M1 pro-inflammatory and neurotoxic phenotype or the alternative M2 immunosuppressive and neuroprotective phenotype.^216^ However, researchers have called into doubt the M1 and M2 classifications.^217^ Through snRNA-seq analysis of frozen midbrain tissue from PD patients and controls, seven distinct microglia subpopulations were identified based on the expression of specific marker genes. Among these, the three largest subpopulations were defined by a high expression of P2RY12, HSP90AA1, and GPNMB. The microglial activation trajectory extends from P2RY12^high^ cells to two activation branches, one with highly expressed HSP90AA1 or IL1B241 cells and the other containing GPNMB^high^ cells.^215^ Using scRNA-seq and immunofluorescence analyses in a murine model, the researchers identified a distinct subset of microglia in the midbrain that displayed an intrinsic transcriptional immune alerted signature. Notably, some genes, including *Casp4, Ccl4, Cd83*, and *H2-ab1*, were exclusively overexpressed in the immune alerted subset. Interestingly, this subset was absent in other brain regions, such as the striatum. Furthermore, the study found that the microglia in the midbrain had a decreased complexity compared to those in the striatum, showing that midbrain microglia had a muted reaction to an inflammatory stimulus, displaying a tolerogenic rather than primed phenotype.^218^ In addition, a specific type of microglia, known as Cspg4-expressing microglia, has been identified as having the ability to proliferate triggered by pathological α-syn during neurodegeneration, particularly in PD.^219^ Overall, these findings provide valuable insights into the heterogeneity of microglia in the nigrostriatal pathway and their potential contributions to PD.

Microglia become reactive, migrate to the damaged sites, produce pro- and anti-inflammatory substances, and may phagocytose cellular debris. The microglial activation and recruitment (microgliosis) process is accompanied by increased cytokine levels.^220^ Elevated levels of cytokines (including IL1β, IL2, IL6, IFNγ, and TNFα) and CD4+ lymphocytes have been detected in both serum and CSF of PD patients.^221,222^ By analyzing snRNA-seq data from postmortem midbrain samples of PD patients and controls, the researchers observed an increased number in reactive microglia and a reduction in oligodendrocyte numbers in PD patients. The study also revealed that the reactive microglia states are enriched in cytokine secretion and the stress response to unfolded protein pathways.^215^ A higher level of NLRP3 inflammasome has been observed in microglial cells in the SN of PD patients, which has been linked to the secretion of pro-inflammatory cytokines associated with neurodegeneration.^223,224^

Peripheral inflammation can also affect the brain through the blood-brain barrier or the autonomic nervous system, triggering microglial activation and contributing to neurodegeneration.^225^ The exact contribution of peripheral immune activation versus recruitment and infiltration to this process remains unclear.^226^ Monocytes and macrophages have been found to infiltrate the inflamed brain. Increased expression of proteins associated with non-microglia myeloid cells, such as CD163, has been observed in the brains of PD patients.^227^ An increase in the macrophages, specifically CD163^+^ macrophages was also detected in the area of neurodegeneration in rodent PD models.^228^ The CCL2-CCR2 axis has been implicated in the infiltration of monocytes into the inflamed brain.^229^ Studies in PD mouse models and patients have shown upregulation and activation of CCR2, suggesting a detrimental role of infiltrating monocytes in PD. Additionally, differences in CCL2 levels in serum or CSF have been associated with different clinical subtypes of PD.^230^ Overall, CD163^+^ and CCR2^+^ monocytes appear to contribute to neurodegeneration in PD both through peripheral actions and infiltrating the brain. Compared to healthy controls, PD patients had increased frequencies of Th1 cells and higher levels of IL10 and IL17A in their serum. However, microglial activation in the brain of PD patients was not significantly associated with peripheral inflammation markers. These findings suggest that peripheral adaptive immunity might indirectly contribute to microglial activation during the neurodegenerative process in PD.^231^

#### Microglial activation induced by neurotoxins

The byproduct of synthetic heroin, MPTP, could be taken up by dopaminergic neurons and induces parkinsonism in humans, non-human primates, and mice. Astrocytes convert MPTP into MPP^+^. Dopamine receptors in neurons take up MPP^+^, which inhibits the mitochondrial complex 1 of the electron transport chain, resulting in ATP depletion and oxidative stress. Ultimately, this leads to the death of dopaminergic cells and the activation of pro-inflammatory microglia.^232^ Remarkably, reactive M1 microglia were found several years after MPTP exposure in humans and non-human primates exposed to MPTP, indicating a long-lasting reactive microgliosis.^233^ In rats, 6-OHDA injection also causes reactive microgliosis, which precedes astrogliosis and dopaminergic cell death.^234^ Additionally, LPS/IFN-*γ* induces microglial activation, increases exosome release,^235^ and microglia-derived exosomes facilitate the transmission of α-syn to neurons, accelerating neuronal death.^236^

#### Microglial activation and phagocytosis induced by α-syn

Microglia have pattern recognition receptors (PRRs) such as TLRs, nucleotide-binding oligomerization domain (NOD)-like receptors (NLRs), and scavenger receptors (SRs) that enable them to detect and respond to different stimuli, including extracellular α-syn. When α-syn accumulates outside of cells and is not properly cleared, it can activate microglia through PRRs, leading to the release of inflammatory cytokines. Chronic inflammation and neuronal damage contribute to the development of PD.^9^ Remarkably, neuroinflammation induced by α-syn may occur even before the loss of dopaminergic neurons in PD. Production of neuroinflammation may trigger α-syn oligomerization, leading to a harmful cycle of microglial activation.^237^

Subsequent investigations of postmortem brain tissue from PD patients revealed that reactive microglia, characterized by amoeboid-shaped morphology, were associated with α-syn pathology in the SN and hippocampus.^220^ α-syn is primarily located at presynaptic terminals and can be released by neurons through different mechanisms depending on its form. Monomers of α-syn are released passively through compromised cell membranes, while aggregated α-syn is released through non-classical exocytosis or multivesicular bodies. As CNS monitors, microglia take up and degrade α-syn, whose internalization can activate microglia and cause neuroinflammation.^238,239^ Different conformation states of α-syn can activate microglia in PD, leading to increased secretion of IL-6, IL-1β, and NO.^240^

**Fig. 3:**
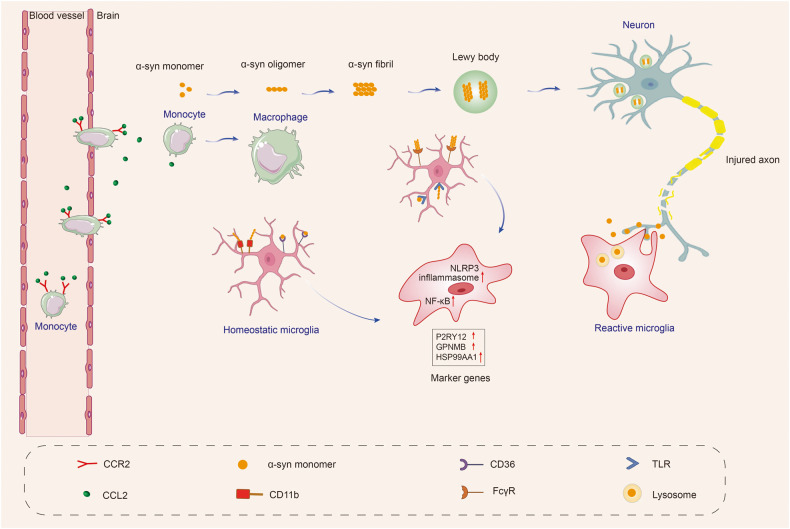
α-syn resulting in microglial response in Parkinson’s disease. Microglia are activated by α-syn, which can be encountered through phagocytosis of synapses or exocytosis from neighboring neurons. Different forms of α-syn, including monomeric, oligomeric, and fibrillar, can be encountered as the disease progresses. The recognition, uptake, and phagocytosis of α-syn by microglia are dependent on the type of α-syn encountered and the involved receptors and proteins. α-syn has been shown to initiate a pro-inflammatory response by interacting with membrane receptors that activate NF-κB through various mediators and assemble the NLRP3 inflammasome, leading to the production of inflammatory mediators and free radicals. The CCL2-CCR2 axis is involved in the infiltration of monocytes into the inflamed brain. Upregulation and activation of CCR2 have been observed in PD mouse models and patients, indicating a potentially harmful role in infiltrating monocytes in PD. Local cytokine and tissue signals can then induce the transformation of monocytes to macrophages. These cascades also result in the proliferation and migration of microglia. Created with https://BioRender.com

Microglia have a high phagocytic capacity. The phagocytic ability of microglia is crucial for α-syn clearance and thus plays a role in the development of PD.^241^ In PD, α-syn is overexpressed and aggregates into oligomers or protofibrils, which can propagate between cells and disrupt the electrophysiological properties of synapses. These aggregates also act as chemoattractants, directing microglia toward damaged neurons. α-syn overexpression has been shown to drive microglia polarization towards a pro-inflammatory phenotype, resulting in increased production of inflammatory cytokines such as IL-1β, IL-6, and TNF-α, as well as enzymes such as COX-2 and iNOS, and the generation of free radicals.^242,243^ TLRs, TAM receptors (Tyro 3, Axl, and Mer), scavenger receptors CD14, and TREM-2 all play roles in microglial phagocytosis.^81,244,245^ TLRs, including TLR2 and TLR4, are receptors expressed on microglia, which were demonstrated to play a role in the uptake of α-syn and the subsequent activation of microglia, boosting α-syn clearance by microglia. Oligomeric α-syn directly engages TLR1/2 and TLR4 to induce a pro-inflammatory M1 phenotype.^246,247^ CD36 and P2X7 receptors are also involved in microglial activation induced by α-syn.^248,249^ α-syn-triggered phagocytic activity of microglia is critical for α-syn clearance and prevents accumulation of misfolded α-syn. Extracellular α-syn is taken up by microglia, perhaps via the autophagy receptor p62, and degraded by selective autophagy.^239^ Although glia can be beneficial in clearing misfolded aggregates in normal physiological conditions, this mechanism may have negative consequences in disease. The uptake and processing of non-toxic α-syn by glia could lead to the generation of toxic strains specific to the disease through autophagy and impaired lysosomal degradation^250^ (Fig. 3).

#### Crosstalk between microglia and neuron in PD

Several studies have revealed that the CX3CR1 receptor expressed on microglia is critical in neuron-microglia communication. This receptor specifically recognizes the protein CX3CL1.^251^ Studies have suggested that the CX3CL1-CX3CR1 signaling pathway is essential for maintaining a healthy balance in microglial activity, regulating chemoattraction and synaptic plasticity, and reducing microglia-mediated inflammation and neurotoxicity.^252^ Another protein that regulates microglial activation is CD200, which is expressed on the surface of neurons and interacts with microglial CD200R. Dysregulation of the CD200-CD200R pathway has been linked to increased microglial activation and degeneration of dopaminergic neurons.^253^ Besides, CB1 receptors are abundant in neurons, while CB2 receptors are primarily expressed in microglia in the brain. Several animal models of PD show that microglial CB2 receptor activation is neuroprotective and improves motor symptoms.^254,255^

#### Genetic mutations and microglial cell activity in PD

Among the genes linked to familial PD, some are involved in inflammatory processes. For example, mutations in SNCA have been proposed to cause aberrant conformation in α-syn, resulting in enhanced conversion of soluble α-syn into insoluble aggregates identified in PD.^256^ Extracellular α-syn exposure increases pro-inflammatory cytokine production in microglial cells.

LRRK2 (PARK8) gene mutations are the most common genetic cause of both familial and sporadic PD.^257^ Previous studies have shown that LRRK2 expression is particularly high in various immune cells, including microglia, macrophages, and monocytes. In contrast, its expression in T cells is relatively low.^258,259^ These findings suggest that LRRK2 may primarily modulate the innate immune system and inflammation in PD. Research on microglia has shown that TLR2 or TLR4 stimulation can increase the expression and phosphorylation of LRRK2.^259,260^ The researchers identified microglia-specific chromatin regions that regulated LRRK2 expression and showed that a specific regulatory DNA element containing the PD-associated genetic variant rs6581593 modulates LRRK2 expression in microglia. These findings highlight the importance of considering cell type when studying the impact of non-coding genetic variants on disease pathogenesis and provide mechanistic insight into the association between the 5’ region of LRRK2 and PD risk.^261^ Furthermore, inhibition of LRRK2 kinase activity can lead to the phosphorylation of NF-κB inhibitory subunit p50 at the protein kinase A (PKA) -specific phosphorylation site S337. This results in an abnormally higher proportion of nuclear P-p50, which may inhibit the function of NF-κB, preventing efficient DNA binding and gene transcription activation in response to inflammation.^262^

PARK7 encodes a tiny peptidase protein known as DJ-1. DJ-1 dysfunction is implicated in a small percentage (1–2%) of inherited forms of early-onset PD.^256^ In microglia, the knockdown of DJ-1 has been shown to enhance the production of inflammatory cytokines in response to LPS.^263^ Additionally, DJ-1 may function as a scaffold protein facilitating the interaction between signal-transducers and activators of transcription (STAT1) and its phosphatase, Src-homology 2-domain containing protein tyrosine phosphatase-1 (SHP-1) that negatively regulates inflammatory responses of microglia. In DJ-1 KO mice, microglia exhibited increased expression of phosphorylation levels of STAT1, as well as inflammatory mediators COX-2, iNOS, and TNF-α.^264^ In addition, microglia lacking DJ-1 showed increased mitochondrial activity, leading to elevated levels of ROS compared to normal microglia, and this effect was further increased by treatment with LPS.^265^

The investigation of genetic mutations associated with microglia significantly enhances our comprehension of the cellular pathways involved in PD. By unraveling these molecular mechanisms, we gain valuable insights into the underlying causes and potential therapeutic targets by modulating specific genes to restore or modify microglia function.

### Multiple system atrophy

Multiple system atrophy (MSA) is a rare, progressive, and fatal neurodegenerative disease characterized by autonomic dysfunction, parkinsonism, and cerebellar ataxia.^266^ Incidence rates are estimated at 0.6–0.7 cases per 100,000 person-years.^267^ Depending on the predominant symptom, MSA is clinically classified into two subtypes: MSA with predominant cerebellar ataxia (MSA-C) and MSA with predominant parkinsonism (MSA-P), whereas olivopontocerebellar atrophy and striatonigral degeneration represent pathological variants, respectively.^268^ Many MSA patients, however, show a combination of both types.^269^ MSA is an adult-onset disorder; the onset age is 56 ± 9 years old.^270^ In MSA, most patients die within 6 to 10 years of diagnosis.^266^ The pathological hallmark of MSA is the abnormal accumulation of α-syn in the cytoplasm of oligodendrocytes, named glial cytoplasmic inclusions (GCIs).^271–275^ Mechanismly, various factors including the abnormal accumulation of α-syn, microglial activation and neuroinflammation,^276^ autophagic impairment,^277–279^ mitochondrial,^280^ and proteasomal dysfunction^281,282^ are involved in the pathogenesis of MSA.

#### Microglial activation in MSA brains

The exact pathogenesis of MSA remains a mystery, despite the suspicion of several players contributing to neurodegeneration. But according to pathological studies and PET imaging of MSA brains, microglial activation and neuroinflammation constitute important features of MSA.^283–287^ Histopathologically, microglial activation was found to be prominent in regions of motor-related structures, including cerebellar input, extrapyramidal and pyramidal motor structures, demonstrating that the mode of microglial activation was consistent with the known pattern of MSA-specific system degeneration,^283^ which suggests that microglial activation likely promotes neurodegeneration in MSA. In addition, the stereology method was used to estimate the number of neurons and glial cells (microglia, oligodendrocytes, astrocytes) in the neocortex of 11 MSA and 11 controls^284^ and white matter of 10 MSA and 11 controls.^285^ The results showed significant widespread microgliosis in both neocortex and white matter in MSA patients compared with controls.^284,285^

PET imaging also demonstrated microglial activation in the brains of patients with MSA. In a study utilizing [^11^C](R)-PK11195 PET imaging to localize microglial activation in 14 MSA patients and 10 controls, it was found that MSA-P patients had significantly higher binding potentials in the precentral gyrus, caudate nucleus, putamen, pallidum, orbitofrontal cortex, superior parietal gyrus and presubgenual anterior cingulate cortex than controls.^286^ Notably, the mean disease duration of these MSA-P patients was 2.9 years (range 2–5 years),^286^ suggesting widespread microglial activation occurs early in the clinical stage of MSA. Interestingly, a recent study using [^11^C] PBR28 PET imaging compared the pattern of microglial activation between 66 MSA (30 MSA-P and 36 MSA-C) and 24 PD patients and observed a conspicuous pattern of increased microglial activation in the cerebellar white matter and lentiform nucleus in MSA compared with PD.^287^ This pattern by visual reading achieved 100% specificity and 83% sensitivity in discriminating MSA from PD,^287^ suggesting that microglial activation has a specific pattern in MSA, although microglial activation has been considered a common immune response in neurodegenerative diseases.^288^

#### Microglial activation is associated with α-syn deposition

The neuropathological hallmark lesion of MSA is the presence of GCIs, located in oligodendrocytes.^289^ A significant constituent of GCI is insoluble α-syn. Evidence is still lacking concerning whether microglial activation precedes the emergence of GCIs. However, α-syn species are believed to activate microglial cells, accelerating neurodegeneration in MSA.^290^ Histopathological studies showed that microglial activation increased in regions with a high α-syn load in MSA patients.^291^ For example, microglial activation was evident in white matter where α-syn inclusions were abundantly observed.^291^ Transgenetic mouse models of MSA overexpressing α-syn in oligodendrocytes also presented early and significant microglial activation and related neuroinflammation accompanying the α-syn accumulation in oligodendrocytes.^292,293^ However, α-syn aggregation, microglial activation, and neuronal death usually coexist in the same brain regions, so does microglial activation secondary to α-syn aggregation or neuronal loss? Several studies exposed primary microglia to α-syn monomers or high-ordered oligomers. They found that high-ordered oligomers rather than monomers induced microglial activation identified by both morphological changes from bipolar to amoeboid and biochemical activation profiles manifested with increased pro-inflammatory cytokines secretion.^294^ The α-syn-induced microglial activation depends on TLRs 1/2 signaling^294^ and TLR4 signaling.^246^ The above evidence indicates that via a specific interaction with TLRs, the misfolded α-syn directly activates microglia and promotes the production and release of pro-inflammatory cytokines.

#### Microglial roles in the pathogenesis of MSA

##### Phagocytosis impairment

**Fig. 4:**
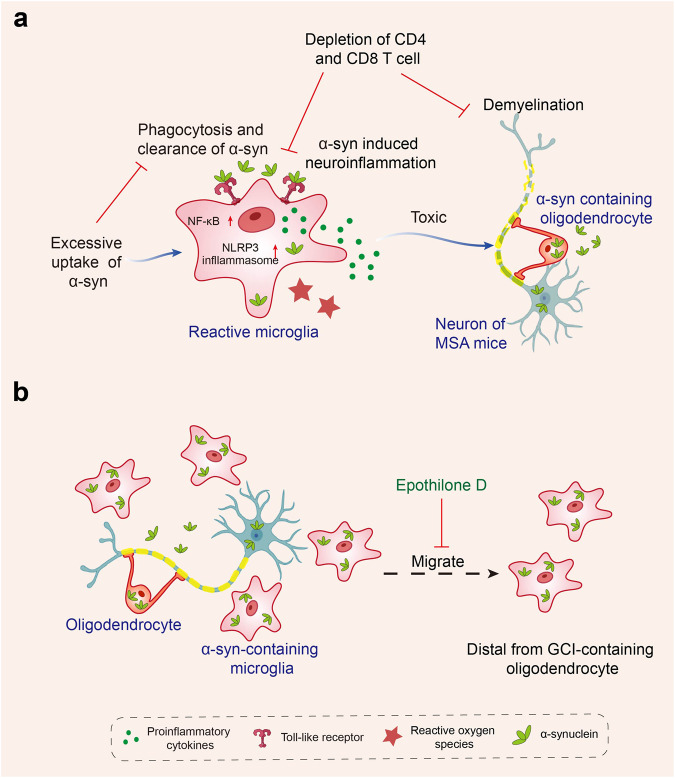
Microglial roles in the pathogenesis of multiple system atrophy. **a** α-syn can interact with microglial toll-like receptors (TLRs) and then was phagocytosed by microglia in an MSA mouse model. But excessive uptake of α-syn by microglia led to a significant decrease in the phagocytic ability of microglia, triggered an inflammatory response of microglia including NF-κB and NLRP3 inflammasome signaling activation, reactive oxygen species production, pro-inflammatory cytokines upregulation, and eventually induced neurodegeneration. Besides, CD4 and CD8 T cell depletion attenuated α-syn-induced inflammation and demyelination in MSA mice. **b** Microglia act as a mobile vehicle to propagate α-syn after phagocytosis of α-syn in MSA patients. An in vitro study showed the ability of microglia to transport α-syn distally was impaired when treated with Epothilone D, a natural product that can inhibit microtubule activity. Created with https://BioRender.com

TLR4-mediated microglial phagocytosis of α-syn in MSA. *TLR4* gene ablation in a transgenic mouse model of MSA with oligodendroglial α-syn overexpression impaired the phagocytic ability of microglia to α-syn, leading to enhanced motor impairment and augmented loss of nigrostriatal dopaminergic neurons.^295^ In contrast, TLR stimulation with a TLR4 selective agonist (monophosphoryl lipid A) in the same MSA mouse model ameliorated motor deficits and rescued nigral neurodegeneration.^245^ In MSA brains, increased brain levels of α-syn were linked to disturbed TLR4-mediated microglial phagocytosis of α-syn.^295^ Evidence consistently showed that microglia could phagocytize α-syn, but excessive uptake of α-syn by microglia led to neurodegeneration. A study generated a mouse model by lentiviral-mediated selective α-syn accumulation in microglial cells in substantia nigra and found that these mice developed progressive degeneration of dopaminergic neurons. Mechanistic studies have found that α-syn aggregated in microglia led to a significant decrease in the phagocytic ability of microglia and triggered an inflammatory response, increasing the release of pro-inflammatory factors, ROS, and nitric oxide by microglia, which created a toxic environment eventually led to neurodegeneration^295^ (Fig. 4).

##### Neuroinflammation

TLR4-mediated α-syn phagocytosis by microglia^245,246,295^ and induced NF-κB translocation, ROS production, and the release of pro-inflammatory cytokines such as TNF-α.^246,296^ Besides, the activation of microglia by α-syn was also dependent on TLR1/2 signaling,^294^ suggesting a modulatory role of TLRs on α-syn induced microglial pro-inflammatory responses and ROS release. Thus, misbalanced TLRs signaling may be crucial for MSA progression. Significantly increased levels of TLR-4 and TLR-1 were observed in multiple brain regions, such as substantia nigra and striatum, in MSA patients versus controls.^297^ Upregulated TLRs are a double-edged sword. It may have a protective effect by increasing the phagocytosis of α-syn by microglia, but as a result, it induces neuroinflammation and promotes neurodegeneration. Besides, NLRP3 inflammasome is probably involved in neuroinflammation in MSA. It triggers pyroptotic cell death in microglia and astrocytes by producing IL-1β and IL-18 pro-inflammatory cytokines.^298^ Immunohistochemical staining of postmortem brains found that the NLRP3 inflammasome was significantly upregulated compared with controls and was significantly correlated with the deposition of GCIs and neurodegeneration in the putamen of MSA^299^ (Fig. 4). The above evidence suggests that the NLRP3 inflammasome may be related to neurodegeneration in MSA. Whether and how the NLRP3 inflammasome participates in MSA pathogenesis needs further research.

##### Propagation of α-syn

A recent study observed the brain tissue sections of MSA patients by immunofluorescence and found that some α-syn-containing microglia were distal from GCI-containing oligodendrocytes,^300^ so it was speculated that these microglia acted as a mobile vehicle to propagate α-syn in MSA after uptaking of α-syn instead of degrading these misfolded proteins in place.^300^ In vitro experiments confirmed this hypothesis. In an in vitro culture system, α-syn was immobilized in the center of a glass coverslip and then treated either with microglia-like differentiated THP-1 cells or undifferentiated THP-1 cells. The proportion of α-syn-containing differentiated THP-1 cells was significantly increased in distant regions of α-syn compared with undifferentiated cells.^300^ The ability of microglia to transport α-syn distally was impaired when microtubule activity was inhibited with Epothilone D (EpoD)^300^(Fig. 4). Therefore, it is proposed that inhibiting microglial migration may be one of the therapeutic strategies to reduce the α-syn spreading.^300^ However, blocking the migration of microglia may also weaken microglial physiological function. Thus, specifically inhibiting the migration of α-syn transporting cells may offer a more effective and promising therapeutic approach in the future.

##### T cell involvement

T cell infiltration alongside the inflammatory microgliosis was observed both in postmortem brain tissues of MSA patients and in a virus-mediated mouse model of MSA.^301^ Meanwhile, in the same MSA mouse model, the proportion of Th1 T cells and the level of INF-γ cytokine in the striatal tissue were significantly increased.^301^ Moreover, MSA mice with Tcrb (CD4 and CD8 T cell knockout) or Cd4 (CD4 T cell knockout) genetic deletion showed reduced inflammation and demyelination after exposure to syn^301^ (Fig. 4). Therefore, a hypothesis is proposed: α-syn in oligodendrocytes induces microglial activation, and then the microglial signals trigger CD4 + T cell infiltration. After CD4^+^ T cells infiltrate the CNS, their T cell receptor binds to the upregulated MHCII on the surface of microglia, which promotes the differentiation of CD4 T cells into Th1 T cells and secretes INF-γ. All these pro-inflammatory cells, including microglia and CD4 T cells, cause oligodendrocyte dysfunction and striatal and corpus callosum demyelination. These results suggest that T cell infiltration into the CNS and their interaction with microglia are key mechanisms of disease pathogenesis in MSA.^301^

### Amyotrophic lateral sclerosis and frontotemporal dementia

Amyotrophic Lateral Sclerosis (ALS) is characterized by the loss of motor neurons in the cortex, brainstem, and spinal anterior horn. The median survival time from the onset of symptoms for ALS is 30 months.^302^ Mutation in *C9orf72* accounts for 40% of familial ALS and FTD cases.^303,304^ Mutations in the SOD1 gene (encoding superoxide dismutase 1) occur in ~20% of cases,^305^ while mutations in TARDBP (encoding TAR DNA- binding protein 43 [TDP43])^306^ and FUS (encoding the fused in sarcoma protein) have a lower frequency (<5%).^307^ Postmortem studies^308–310^ and in vivo PET studies^311–313^ revealed widespread cerebral microglial activation in patients with ALS,^308–312^ and even in pre-symptomatic ALS-related mutation carriers.^309,313^ Microglial activation in the corticospinal tract of ALS correlated with neuronal and axonal loss and more severe upper motor neuron symptoms.^309^ Microglial activation in middle frontal and superior or middle temporal gyrus regions was also highly found in vivo PET imaging studies in humans, indicating a pivotal role for microglia in ALS. However, whether the involvement of microglial activation is beneficial or detrimental remains unclear. Therefore, the researchers employed various transgenic mouse models to study the functional or pathological role of microglia in ALS pathogenesis.

Frontotemporal dementia (FTD) is the second most common form of dementia after AD in people among people under 65, and is characterized by progressive cognitive, behavioral, and language dysfunction. Pathological studies have shown the deposition of abnormal proteins in the brain tissues of FTD patients, including TDP-43 (50% of cases), tau (40% of cases), and FUS (5–10% of cases).^314,315^ Gene mutation is an important pathogenic factor for FTD. It is estimated that 60% of patients with FTD have autosomal dominant mutations in *C9orf72*, *GRN*, or *MAPT* genes.^316^ Additionally, mutations in *TBK1* are associated with developing FTD,^317,318^ and variants in *TREM2* increase an individual’s risk of developing FTD.^319^ In vivo PET studies^320,321^ and immunohistochemistry studies^322–324^ have found increased microglial activation in the frontal and temporal cortices of patients with FTD.

#### Introduction of commonly used animal models of ALS and FTD

Several experimental animal models have been established, given the pathogenic mutations found in ALS and FTD. The human SOD1 mutant mice (mSOD1) are widely used as ALS models because they exhibit progressive paralysis, pathological protein aggregation, motor neuron degeneration, and gliosis, which are hallmarks of ALS patients.^325–327^ However, neuron-specific expression of mSOD1 in transgenic mice failed to produce pathology or disease in these mice,^328,329^ suggesting a role of non-cell-autonomous factors, possibly from non-neuronal cells such as microglia, in ALS pathogenesis. Microglial activation was observed in SOD1 models, and microglial activation was detected before the onset of clinical disease in some studies.^330,331^ Several studies have demonstrated the role of microglia in ALS mSOD1 mouse models.

Ninety-seven percent of patients with ALS and 50% of patients with FTD have cytoplasmic TDP-43 aggregates within neurons,^332^ and mutations in TARDBP, the gene encoding TDP-43, have been identified in patients with ALS or FTD,^306,333^ indicating a mechanistic link between TDP-43 and ALS/FTD and also suggesting a common underlying mechanism, which prompts the use of TDP-43 mutant transgenic mice to study TDP-43-related neurodegeneration in ALS and FTD. In TDP-43 mouse models, pathogenesis is involved in a combination of loss of nuclear TDP43 function and TDP-43 cytoplasmic gain-of-toxic-function.^334^

*C9orf72* intronic hexanucleotide repeat expansion (GGGGCC) was identified as the most common genetic cause of FTD and ALS.^304,335^ The mechanism by which these repeat expansions may cause disease is outlined in three hypotheses: first, downregulation of gene expression (haploinsufficiency) leads to a loss of *C9orf72*’s normal cellular function; second, the presence of sense and antisense RNA foci could sequester RNA-binding proteins, resulting in splicing defects in RNA metabolism; and third, the formation of repeat-associated non-ATG-translated dipeptide repeat proteins (DPRs) may cause neurodegeneration due to gain-of-function toxicity.^336^ Tansgenetic mouse models have been used to examine the role of *C9orf72* and its hexanucleotide repeat expansion in the pathogenesis of FTD and ALS, and the results suggest that the microglia are involved in *C9orf72*-mediated ALS/FTD pathogenesis.

Heterozygous GRN mutations cause the deficiency in progranulin (PGRN), a lysosomal and secreted protein, and contribute to the development of FTD,^337^ while the homozygous GRN mutation leads to neuronal ceroid lipofuscinosis.^338^ PGRN is expressed in microglia and neurons and shows higher expression once microglia respond to the changes in their CNS environment. Grn loss of function causes microglial activation,^339,340^ cytosolic phosphorylated TDP-43 aggregation,^339,340^ myelin debris accumulation in white matter, and spatial learning and memory impairment in *Grn* ^-/-^ mice.^340^

#### Microglial roles in pathogenesis of ALS and FTD

##### Phagocytosis of microglia

Microglia could phagocytize mobile TDP-43 deposits within degenerating motor neurons.^341^ There is also evidence that microglia actively facilitate the clearance of hTDP-43 in a transgenic TDP-43 mouse model.^342^ Nonsense or missense mutations in *TREM2* have been identified in several families with FTD.^343–345^ One study comprising 609 patients with FTD and 1957 controls found that the *TREM2* p.R47H variant is a risk factor for FTD (OR = 5.06; *p*-value = 0.001).^346^ Homozygous *TREM2* p.T66M mutation has also been identified to cause FTD-like syndrome.^344^ Recently, Xie et al. found that TDP-43 interacted with microglial TREM2 and induced TREM2-dependent microglia with phagocytic ability that facilitated clearance of TDP-43.^347^ In mice lacking TREM2, microglia were locked in a homeostatic state, and TDP-43 clearance by microglia was reduced, leading to increased neuronal damage and motor impairments.^347^ This finding may partly explain the link between the *TREM2* mutant and the FTD-like manifestation.

In the CNS, *Grn* loss of function profoundly impacts the microglial state and function, which plays a critical role in FTD pathogenesis. Deficiency of *Grn* caused synaptic loss by microglia-mediated synaptic pruning via complement involvement as deletion of the C1qa gene (which encodes complement C1q subcomponent subunit A) significantly reduced synaptic pruning by *Grn*^-/-^microglia, and ameliorated behavioral deficits, neuronal cell loss and increased survival in *Grn*^-/-^ mice.^348^ *Grn* deficiency led to microglial lysosomal dysfunction and a reduced ability to degrade myelin debris, thus leading to the accumulation of myelin debris in microglial lysosomes and increased microgliosis and myelin debris accumulation in the white matter of *Grn*^−/−^ mice and patients with GRN-associated FTD.^349^

Overall, microglia could phagocytize pathological protein deposits such as TDP-43, and the interaction of TDP-43 with microglial TREM2 promotes the phagocytosis and clearance of TDP-43 by microglia. In the disease state, microglial lysosomes undergo dysfunction, which reduces the clearance of deposited protein and myelin debris and accumulates myelin debris in the white matter. In addition, microglia increase synaptic pruning through interaction with complement, which exacerbates neuronal loss and behavioral abnormalities. The above dysfunctional modulation will promote the occurrence and development of the disease.

##### Neuroinflammation

TDP-43 protein could be secreted into the extracellular matrix via exosomes^350^ or cell death. Increased levels of TDP-43 in CSF have been detected in patients with ALS^351,352^ and FTD.^352^ In vitro studies found when WT or various forms of mutant TDP43 protein or TDP43 aggregates were added to primary microglial cultures, they were internalized, leading to microglial activation, NLRP3 inflammasome and pro-inflammatory markers upregulation, in which mutant TDP43 eliciting a more pronounced response.^353^ These TDP-43 proteins or aggregates-induced microglial activation and neuroinflammation have neurotoxic effects on co-cultured motor neurons.^354^ Notably, in the absence of microglia, added TDP-43 proteins were not toxic to cultured primary motor neurons,^354^ suggesting the involvement of non-cell autonomous pro-inflammatory effects mediated by microglia that enhance motor neuron injury. Therefore, in the context of the TDP-43 mouse model, microglia can phagocytize pathological TDP-43 to play a protective role and drive neuroinflammation, resulting in neuronal damage.

The anti-inflammatory cytokine IL-10 was upregulated in microglia at pre-symptomatic stages in SOD1 mice. In vitro studies found that upregulated IL-10 delayed the occurrence of the disease.^355^ Another study also found that microglia presented an anti-inflammatory phenotype in SOD1^G93A^ mice at disease onset and were neuroprotective to motor neurons. In contrast, microglia presented a pro-inflammatory phenotype at end-stage disease and were neurotoxic to motor neurons.^356^ Reduced mutant levels in microglia did not have much effect on early disease progression, but substantially slowed it later on,^306^ emphasizing that microglia in the late stage accelerate disease progression. These findings suggest that modulating microglial phenotype to avoid its conversion to a pro-inflammatory state during disease progression will be helpful for the treatment of ALS.

*Grn* deficiency leads to NF-κB activation in microglia and elevation of TNFα, leading to hyperexcitability of medium spiny neurons and obsessive-compulsive disorder-like behavior.^357^ Recently, interleukin-18 receptor accessory protein (IL18RAP) 3′ untranslated region (3′UTR) variants were identified as protective factors which reduced the risk of developing ALS fivefold, delayed onset, and therefore the age of death in individuals with ALS. The 3′ untranslated region (3′UTR) variants of interleukin-18 receptor accessory protein (IL18RAP) has been identified as protective factors in ALS patients. They reduced the risk of developing ALS fivefold.^358^ These variants in the IL18RAP 3′UTR reduce mRNA stability and the binding of double-stranded RNA (dsRNA)-binding proteins. Mechanistically, IL18RAP is upstream of NF-κB; thus, the variants downregulate IL18RAP–NF-κB signaling in microglia. This study highlights the protective effect of alleviating microglia-mediated neurotoxicity in ALS.

##### Phenotype switching of microglia contributes to disease progression

Homeostatic microglia are protective against ALS. Antibiotic administration to SOD1 mice altered gut microbiota composition, weakened motor function, and caused death in the SOD1 mice. There was a downregulation of homeostatic genes and an increase in neurodegenerative genes in microglia after antibiotic treatment, and the microglia signature change preceded changes in motor function.^359^ Grn loss of function promoted the microglial transition from a homeostatic to a disease-specific state, aggravated TDP-43 pathology and neurodegeneration through the synergy of microglia and the complement system since deleting the genes for C1qa and C3 mitigated microglial toxicity and rescued TDP-43 proteinopathy and neurodegeneration.^360^

Microglia showed higher levels of *C9orf72* expression than other cell types in the brain,^361^ and research suggests that *C9orf72* is required for the normal immune function of myeloid cells. In the *C9orf72*^–/–^ mice, progressive splenomegaly, lymphadenopathy, and upregulation of pro-inflammatory cytokines were observed at the periphery, while brain and spinal cord microglia showed an inflammatory state.^361^ *C9orf72* is important for maintaining microglia homeostasis. Transcriptome and histologic analyses of postmortem tissue from patients with ALS carrying the *C9orf72* expansion (C9-ALS) found that decreased C9orf72 expression in C9-ALS led to altered microglial function and increased microglial activation.^309,361^ In Lall et al.’s study, Cd11b+ isolated microglia from young (3 months) and aged (17 months) *C9orf72*^+/+^, *C9orf72*^+/-^, and *C9orf72*^-/-^ mice were analyzed by RNA-seq. Results showed that loss of *C9orf72* promoted microglial gene signature transition from homeostatic to an inflammatory state characterized by an enhanced type I IFN signature. Co-cultured *C9orf72*^+/+^ or *C9orf72*^-/-^ microglia with WT cortical neurons found that loss of *C9orf72* in microglial cells promoted microglial phagocytosis and neuronal synaptic loss. Thus, the authors argued that microglial impairment from decreased C9ORF72 expression directly contributed to neurodegeneration in C9-ALS.^362^ Although the loss of C9orf72 alone is insufficient to cause ALS motor deficits,^363^ the loss of function of C9ORF72 may lead to microglial dysfunction, which may accelerate disease progression in C9ALS/FTD patients.

##### Peripheral immune cells are involved in the regulation of microglia

**Fig. 5:**
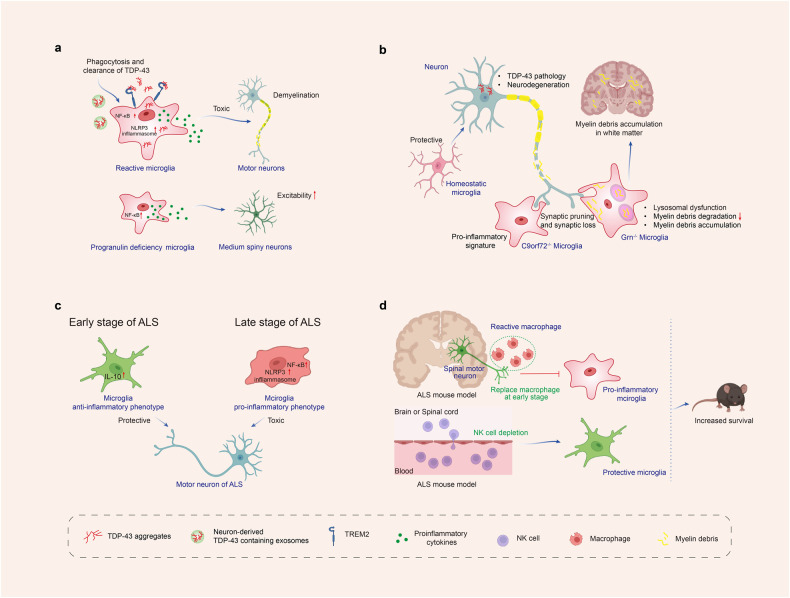
Microglial roles in the pathogenesis of amyotrophic lateral sclerosis and frontotemporal dementia. **a** TDP-43 could be released into the extracellular matrix via exosomes or cell death. TDP-43 interacted with microglial TREM2 and then increased their phagocytosis and clearance by microglia. Phagocytosis of TDP43 aggregates by microglia led to microglial activation, microglial NLRP3 inflammasome activation, and pro-inflammatory markers upregulation, which have neurotoxic effects on motor neurons. Besides, progranulin deficiency in microglia activated microglial NF-κB signaling and promoted the release of pro-inflammatory cytokines, leading to hyperexcitability of medium spiny neurons. **b** Homeostatic microglia are protective against ALS. Progranulin deficiency aggravated TDP-43 pathology, promoted synaptic pruning by microglia, and led to synaptic loss and neurodegeneration. Progranulin deficiency also led to microglial lysosomal dysfunction and reduced myelin debris degradation, leading to the accumulation of myelin debris in white matter. Besides, *C9orf72* loss of function promoted microglial gene signature transition from a homeostatic to an inflammatory state and promoted synaptic pruning by microglia and synapse loss in neurons. **c** At the early stage of the ALS mouse model (SOD1 mice), the anti-inflammatory cytokine IL-10 was upregulated in microglia, and microglia presented with an anti-inflammatory phenotype, which was neuroprotective to motor neurons. But at the late stage, microglia presented with a pro-inflammatory phenotype, which was neurotoxic to motor neurons. **d** Peripheral nerve reactive macrophages along peripheral axons of motor neurons were activated in an ALS mouse model (SOD1 mutant mice). Replacing peripheral nerve macrophages at disease onset reduced microglial pro-inflammatory responses and prolonged survival in these mice. NK cell infiltration into the cerebral motor cortex and spinal cord was observed in an ALS mouse model (SOD1^G93A^). NK cell depletion induced a protective microglial phenotype and increased survival in these mice. Created with https://BioRender.com

In SOD1 mutant mice, reactive macrophages along peripheral axons of motor neurons were observed during disease progression. Replacing peripheral nerve macrophages at disease onset suppressed peripheral macrophage inflammation, reduced microglial pro-inflammatory responses, and prolonged survival in mice.^364^ NK cell infiltration into the spinal cord and cerebral motor cortex was observed in sporadic ALS patients and an ALS mouse model (SOD1^G93A^). NK cell depletion induced a protective microglial phenotype and increased survival in the ALS mouse models (hSOD1^G93A^ and TDP43^A315T^). The above evidence suggests that peripheral immune cells shape microglia into a pro-inflammatory phenotype and accelerate disease progression in ALS^365^ (Fig. 5).

### Progressive supranuclear palsy

Progressive supranuclear palsy (PSP) encompasses a range of clinical phenotypes involving movement, behavioral abnormalities, and language impairments.^366^ PSP has a prevalence rate of around 0.9 per 100,000 person-years.^367^ The average age of onset of PSP is ~68 years, and the survival time after diagnosis is ~2.9 years.^368^ Neuropathologically, PSP is defined by the accumulation of pTau in both neurons and glial cells.^369^ Pathologically pTau leads to depolymerization of microtubules, neuronal damage, microgliosis, and astrogliosis, ultimately leading to irreversible neurodegeneration.^370^ There have been over 30 drugs that target tau aggregation, processing, and accumulation reaching the clinic over the past 15 years, but the results were disappointing.^370^ Recently, GWAS showed that most AD risk genes were highly or exclusively expressed in microglia in the brain such as *TREM2* and *MS4A4A*.^124^ Notably, the expression level of *TREM2* and *MS4A4A* gene were also upregulated in the substantia nigra of PSP patients versus controls. A positive correlation was found between *TREM2* mRNA levels and hyperphosphorylated tau burden in the substantia nigra, specifically in neurons.^371^ The above evidence suggests that microglia participate in the disease process of PSP. Moreover, these findings suggest that tauopathies may share similar immune regulation mechanisms.

#### Microglial activation in PSP brains

Several studies have identified increased microglia number and activation in the substantia nigra pars compacta (SNpc),^372^ subthalamic nucleus,^373^ pyramidal, extrapyramidal motor system, and cerebellar output^374^ of brain tissues from PSP patients by immunochemical postmortem analysis. And in most of these regions, microglial activation was positively correlated with tau burden.^374^ Microglial activation in PSP brains was also detected by PET imaging. PSP patients showed significantly increased mean [^11^C](R)-PK11195 binding in the midbrain, basal ganglia, frontal lobe, cerebellum,^375^ thalamus, putamen, and globus pallidus^376^ compared to controls. Using another TSPO tracer ^18^F-GE-180, elevated TSPO uptake was also observed in subcortical brain regions, especially in the medial pallidus in PSP.^377^ Importantly, by using [^11^C]PK11195 and [^18^F]AV-1451 to measure microglial activation and tau pathology, respectively, studies found that microglial activation in cortical and subcortical regions was positively correlated with tau pathology.^378^ Additionally, subcortical tau pathology and microglial activation positively correlated with clinical severity^376,378,379^ and could predict disease progression in PSP.^379^ Taken together, the above pathological and neuroimaging evidence consistently showed that microglial activation was closely related to tau pathology and neurodegeneration, suggesting that microglia is likely to play a crucial role in the pathogenesis of PSP.

#### Tau serves as a main trigger of microglial activation

It has been hypothesized that tauopathy is the primary cause of microglial activation. Firstly, microglial activation colocalized and correlated with tau pathology in PSP patients^374,378^ and transgenic mice expressing mutant tau P301L.^380^ Secondly, to clarify the temporal sequence of microglial activation and tauopathy, a study used Thy1-hTau.P301S mice that expressed human tau with a P301S mutation, specifically in neurons. From 2 months onward, there was significant cortical pTau deposition, and several changes were observed in microglia following pTau accumulation, including morphological changes, an increase in microglial lysosomal volume, and a significant loss of homeostatic marker Tmem119.^381^ Thirdly, when applied in vitro, overexpression of full-length tau in cultured rat microglia caused their activation.^382^ Alternatively, tau oligomers and fibrils added into the culture of primary microglia directly caused microglial activation and secretion of pro-inflammatory cytokines.^383^ Together, these findings indicate that microglia adopt a pTau-induced phenotype. In vitro study also revealed that MAPK pathway mediated tau-induced microglial activation.^384,385^

#### Microglial roles in PSP pathogenesis

##### Phagocytosis

Microglia could phagocytize different tau species,^386,387^ and this process was partly dependent on microglial fractalkine receptor (CX3CR1) receptor.^388^ The deficiency of the CX3CR1 enhanced tau phosphorylation and aggregation and memory impairment in a transgenic mouse model of tauopathy in which the WT human *MAPT* gene replaced the endogenous mouse *Mapt* gene (hTau mice).^389^ Furthermore, microglia could also phagocytize synapses that contain aggregated tau via synaptic tagging of C1q, leading to a decline in synapse density.^390^ A C1q-blocking antibody could inhibit microglial synapse removal and rescue synapse density in cultured neurons and Tau-P301S mice. Notably, microglia can even phagocytize live neurons containing tau inclusions in addition to free extracellular tau.^391^ Mechanically, microglia are activated when live neurons with tau aggregation expose phosphatidylserines as a “eat-me” signal. This is followed by opsonin milk-fat-globule EGF-factor-8 (MFGE8) and nitric oxide released from the microglia, resulting in the engulfment of the live neuron by the microglia.^391^ Together, microglia could phagocytize free extracellular tau, synapses of neurons containing pTau, and even live neurons containing Tau inclusion. It is currently unclear, however, if this process is detrimental or beneficial. Despite clearing tau aggregation, microglia also lead to the loss of synapses and stressed-but-viable neurons, which disrupt neuronal networks and may lead to cognitive impairments.^392^

##### Neuroinflammation

In parallel with the detection of microglial activation in the brains of PSP patients, upregulation of the expression levels of pro-inflammatory factors was also detected in the substantia nigra, such as IL- 1β.^373^ Microglial activation led to the release of pro-inflammatory factors and induced neuroinflammation, further accelerating the accumulation of hyperphosphorylated tau.^393,394^ Importantly, immunosuppression with FK506 significantly decreased tau pathology and increased lifespan in young P301S Tg mice, thereby demonstrating that microglial activation and neuroinflammation promoted the early progression of tauopathies.^395^ The further study discovered that Cx3cl1 expressed by neurons interacted with the microglial receptor Cx3cr1 and restricted microglia-mediated neuroinflammation. Genetic ablation of *Cx3cl1*^396,397^ or *Cx3cr1*^389,398^ in mouse models of tauopathy enhanced tau hyperphosphorylation, tau pathology, neuroinflammation, and cognitive deficits^389,396–398^ via IL-1/p38 MAPK pathway.^389,397^ Notably, merely transplanting purified microglia derived from hTau*Cx3cr1*^-/-^ mice into the brains of non-transgenic recipient mice could induce tau hyperphosphorylation within the recipient brain. But transplantation of inclusion of IL-1 receptor antagonist together into the recipient brain, microglia-induced tau pathology significantly reduced.^398^ The above evidence suggests that reactive microglia, released pro-inflammatory cytokines, and microglia-mediated neuroinflammation are sufficient to drive tau pathology. This also suggests that inhibition of microglial activation and related neuroinflammation could delay the progression of PSP and other tauopathies.

Besides, after microglial uptake and lysosomal sorting of tau fibrillization, aggregated tau-activated NLRP3-ASC-dependent inflammasomes. ASC deficiency in tau transgenic mice significantly inhibited exogenously seeded and non-exogenously seeded tau pathology. Alternatively, exogenously seededtau pathology was inhibited by chronic intracerebral administration of the NLRP3 inhibitor. These findings indicate that aggregated tau activates the ASC inflammasome via the NLRP3-ASC axis, which exacerbates exogenously seeded and non-exogenously seeded tau pathology in vivo.^399^ The mechanism of action of inflammasomes may be partly because inflammasome activation produces pro-inflammatory cytokines IL-1β and IL-18,^298^ which have been shown to increase the tau hyperphosphorylation and accumulation.^400,401^ The findings highlight that the NLRP3 inflammasome may be a therapeutic target in PSP and other tauopathies.

##### Tau propagation

Tau propagation was likely mediated through synaptic connections.^402^ As a result of injecting mice with adeno-associated virus (AAV) expressing human mutated tau under neuron-specific synapsin-1 promoters into their entorhinal cortex, it was observed that tau spread from the entorhinal cortex to the dentate gyrus 4 weeks after injection.^402^ Microglia may be critical in spreading tau protein since depleting microglia dramatically suppressed tau propagation in this mouse model.^402^ Moreover, microglia are pivotal in spreading tau by releasing tau-containing exosomes.^402,403^ Suppressing exosome synthesis and secretion from microglia may halt exosome-mediated tau propagation. Overall, microglia and their secreted exosomes contribute to tauopathy progression and the inhibition of exosome synthesis or secretion could be a therapeutic target.

##### Senescent glial cells

Cell senescence is involved in the occurrence or progression of neurodegenerative diseases.^404^ In Tau-P301S mice, senescent microglial cells and astrocytes were observed at six months of age. To investigate the role of senescent cells in the development of disease, two methods were used to clear senescent cells: first, by genetically engineering mice so that their senescent cells died after being fed a specific chemical, and second, by using senolytic, genetic approach decreased tau pathology and gliosis, prevented neuronal degeneration and thus improved cognitive function, while pharmacological intervention can also attenuate tau phosphorylation. These results show that senescent glial cells directly contribute to neuronal tau pathology and cognitive impairment. This finding also suggests that senescent glial cells may provide a therapeutic target.^405^

##### T cell involvement

T cell infiltration was observed in the cortex of patients with FTD with a P301L tau mutation and the hippocampus of THY-Tau22 tau transgenic mice. T cells were chronically depleted using specific antibodies to gain insight into the functional role of T cell infiltration in tauopathy pathogenesis. Prevention of hippocampal T cell infiltration in tau transgenic mice rescued spatial memory deficits, despite without modulation of tau pathology,^406^ suggesting that T cell infiltration is involved in the pathological mechanism of tauopathies, although the exact mechanism remains unclear.

### Corticalbasal degeneration

Corticobasal Degeneration (CBD) is a rare, progressive neurodegenerative disease. In most cases, the disease begins between ages 60 and 70. CBD is manifested with parkinsonism, myoclonus, sensation loss on one side or difficulty identifying things by touch, alien limb, speech and language difficulties, and behavioral changes. As CBD symptoms overlap with other more common neurological diseases, corticobasal syndrome is more commonly used by neurologists than CBD.

Similar to PSP, increased microglial activation was also found in CBD brains compared with controls, and that microglial activation in most regions correlated with tau burden.^374^ Although both PSP and CBD brains showed microglial activation, a distinctive pattern of microglial activation and tau pathology was found in CBD and PSP, with CBD showing more pathology in supratentorial structures. In comparison, PSP showed more pathology in infratentorial structures.^374^ Another difference was that only CBD patients demonstrated increased TSPO uptake in additional motor and supplementary motor areas^377^ even though significantly increased mean [^11^C](R)PK11195 binding was observed in subcortical brain regions,^377,407^ especially in the medial pallidus^377^ in both CBD and PSP patients. Besides, the activation of microglia also showed asymmetry in CBD. For example, according to fluorodeoxyglucose (FDG) and [^11^C]PK 11195 PET imaging, a CBD patient with left-sided symptoms showed a marked right hemispheric hypometabolism as well as asymmetric microglial activation in corresponding areas of the right temporal and parietal cortex and basal ganglia.^408^ Although both CBD and PSP belong to tauopathies, the exact pathogenesis remains unclear as to why the regions of pathological tau deposition are different and microglial roles in different tauopathies are also unknown.

### Dementia with lewy bodies

Dementia with Lewy bodies (DLB) is the second most common type of degenerative dementia following AD. DLB accounted for 3.2–7.1% of all dementia cases in the incidence studies.^409^ Clinical characteristics of DLB include cognitive fluctuations, parkinsonism-like motor symptoms, recurrent visual hallucinations, and rapid eye movement sleep behavior disorder (RBD).^410^ The defining pathological features of DLB are the presence of intracellular α-syn aggregates termed Lewy bodies.^411^ However, many DLB cases also exhibit Alzheimer-type pathology in the form of Aβ plaques and tangles of hyper-phosphorylated tau.^412,413^ This AD-type pathology in DLB was associated with global and regional atrophy rates.^414,415^

#### Microglial activation and neuroinflammation in DLB postmortem brains

Several studies have used immunostaining to detect microglial activation in the postmortem brain tissue of DLB patients, and the vast majority of these studies found that DLB patients and non-demented controls had similar microglial activation and neuroinflammation levels,^416–420^ except two studies that examined only 5 cases of DLB each.^421,422^ Among these studies, Amin et al.’s study was based on the largest postmortem cohort consisting of 30 postmortem confirmed DLB cases and 29 matched controls, which performed immunohistochemistry in the cerebral cortex to observe microglial phenotype with several markers including Iba1, HLA-DR, and CD68. Even though immunostaining showed increased neuropathology in DLB, there was no significant difference between groups in microglial activation.^420^ Also, transcriptomic and protein expression analyses of postmortem brain tissue in DLB did not reveal significant microglial activation.^419,423^ Although these studies of human postmortem brain tissue did not demonstrate microglial activation in DLB patients, they consistently showed increased cortical recruitment of T lymphocytes in DLB,^420,424^ suggesting that adaptive immunity may be involved in the pathogenesis of DLB.

#### Early microglial activation detected by in vivo PET imaging

In vivo brain imaging allows the investigation of inflammatory processes in the early stages of the disease and facilitates longitudinal monitoring of microglial activation during disease progression. Two studies found increased microglial activation in the early stage of DLB. One study included 6 DLB patients within a year from the onset (MMSE 24 ± 3.9) and found a significant increase in [^11^C]PK11195 binding in nearly all subcortical and cortical brain regions in DLB patients compared with 11 controls.^425^ To further clarify whether microglial activation is associated with severity of cognitive impairment, a larger study of 19 DLB cases (MMSE 21.9 ± 4.5) was divided into two subgroups by Addenbrooke’s Cognitive Examination Revised (ACE-R)score: nine ‘mild’ cases (ACE-R score>65) and 10 ‘moderate/severe’ cases (ACE-R score≤65), indicating their level of cognitive impairment at the time of their ^11^C-PK11195 PET scan. The mild DLB group showed increased ^11^C-PK11195 binding in the inferior and medial temporal gyri, putamen, cuneus, fusiform gyrus, and inferior frontal gyrus compared with controls.^426^ On the contrary, the moderate/severe DLB group demonstrated generally decreased binding compared with controls.^426^ This study suggests that microglial activation occurs in the early stages of the disease and that as the disease progresses, the microglia phenotype changes. This is consistent with another study using [^3^H]PK11195 and [^3^H]PBR28 PET to observe microglial activation and found that TSPO density in the SN of 5 late-stage DLB brains was significantly reduced compared to 8 controls. The authors of this study proposed that this distinctive pattern of TSPO density change in late stage DLB cases may indicate microglia dystrophy.^427^ This is similar to the results of a previous study, which observed the microglia morphology by immunohistochemistry and found that in the late stage of DLB, there was low microglia density and microglia showed dystrophic phenotype.^418^

Overall, whether using postmortem brain tissue immunostaining which reflects the advanced stage of the disease, or in vivo PET imaging of late-stage DLB or those DLB patients with more severe cognitive impairment, it is consistently found that in the late stage of DLB, microglia are not in an activated state, but instead appear to be dystrophic. In contrast, in the early stage, reactive microglia in an activated state were remarkable. This indicates that microglia show different phenotypes in different stages of DLB. To date, the following questions remain unclear, for example, whether early microglial activation in disease is protective or deleterious. Why does microglial activation diminish in the late stages of the disease? Cortical atrophy, neuropil degeneration, and microglial activation are less prominent in advanced DLB than in AD.^420,428^ Is this related to the fact that AD-like pathology (such as Aβ and tau deposition) in DLB is less significant than in AD? Are differences in the severity of neurodegeneration between AD and DLB related to differences in the degree of microglial activation? In the future, it is essential to conduct more longitudinal studies to observe the dynamic changes in microglial activation during disease progression. If possible, monitoring tau or Aβ or α-syn deposition alongside microglial activation may help elucidate the correlation of microglial activation in DLB with other neuropathology.

### Huntington’s disease

Huntington’s disease (HD) is an inherited neurodegenerative disease that manifests with involuntary choreatic movements and cognitive and behavioral disturbances. HD is a monogenic autosomal dominant disorder caused by the expansion of CAG trinucleotide repeats in the HTT gene. The mutation leads to an abnormally long polyglutamine (polyQ) expansion in the huntingtin (HTT) protein, which confers the protein propensity to misfold.^429^ The length of the CAG repeat is critical for developing HD. Repeats up to 35 in length do not cause HD. People with 36–39 CAG repeats may or may not develop symptoms of HD. CAG repetition lengths over 40 are linked to a definite HD onset during a typical lifespan.^430^ Correlative studies have revealed an inverse relationship between the age of onset and CAG repeat length.^431^ The incidence of HD in the Western population is 4–10 per 100,000 people, and the average age of onset is 40 years.^432^ Pathogenic mutant huntingtin (mHTT) is expressed in various types of neurons in the brain.^433^ It causes selective loss of medium spiny neurons (MSNs) in the striatum, as well as caudate and putamen atrophy.^434^ R6/2 and N171-Q82 mouse models expressing truncated N-terminal segments of HTT are commonly used in research. R6/1 and R6/2 mice express exon 1 of the human HTT gene with approximately 116 CAG repeats and 144–150 CAG repeats, respectively, under the control of the human HTT promoter. Mice expressing truncated HTT (R6/2 and N171–82Q) demonstrate more severe behavioral and neurological abnormalities than transgenic mice that express full-length mutant HTT.^435^

#### Microglial activation in Huntington’s disease

Aggregated mHTT is typically observed as inclusion bodies in the cytoplasm or nucleus,^436^ and N-terminal htt fragments are widely believed to play a role in HD pathogenesis, with smaller N-terminal htt fragments showing greater neurotoxicity than large-sized fragments.^437^ Postmortem examination shows 95 percent of HD patients’ brains had bilateral striatal atrophy.^438^ Indeed, it has been demonstrated that neuron cell death in the cerebral cortex is diverse, explaining the various symptom profiles observed in HD.^439,440^ Reactive gliosis is routinely found in the striatum of HD patients, together with reactive fibrillary astrocytosis and reactive microglia.^441^ In vivo PET investigations have demonstrated significant microglial activation in affected areas of the HD brain, and this pathological phenomenon is more pronounced in severe cases of HD.^442^ ^11^C(R)-PK11195 PET detected microglial activation in HD patients even before the onset of clinical symptoms.^443^ Expression of mHTT in neurons leads to microglial activation in cortico-striatal brain slice and primary neuronal culture models. These reactive microglia tend to cluster along aberrant neurites but do not directly contribute to neuronal degeneration.^444^ Using genome-wide approaches, one study revealed that microglia expressing mHTT exhibited an intrinsic pro-inflammatory gene expression profile even without pro-inflammatory stimuli. Moreover, ex vivo and in vivo studies showed that mHTT-expressing microglia are more toxic to neurons than WT microglia after pro-inflammatory stimuli.^445^

#### Major signaling pathways underlying the microglial activation

Several signaling pathways have been implicated in microglial activation in HD, including the NF-kB pathway, kynurenine pathway, and cannabinoid receptor pathway. By expressing TLRs, microglia can detect and respond to a wide range of external stimuli, including infections, tissue damage, and other sources of inflammation. Upon activation, TLRs trigger a series of signaling pathways that lead to the activation of immune responses. TLRs signaling can activate the downstream NF-kB signaling cascade by the intracellular adaptor protein MyD88, leading to an increase in the production of pro-inflammatory cytokines. It has been shown that soluble mHTT activate IκB Kinase (IKK), triggering the NF-kB signaling pathway,^446^ and leads to the increase in the gene expression of pro-inflammatory cytokines.^447,448^ Furthermore, the reduction of mHTT levels with siRNA improved NF-kB transcriptional dysregulation and reduced pro-inflammatory cytokine production in HD.^449^

Kynurenine pathway metabolic balance influences the function of microglia in HD. Kynurenine 3-monooxygenase (KMO) enzyme, which is predominantly expressed in microglia, catalyzes the conversion of L-kynurenine to 3-hydroxykynurenine (3-HK) in the kynurenine pathway.^450^ R6/2 HD mice treated with the KMO inhibitor JM6 showed a decrease in synaptic degeneration and a longer lifespan by reducing microglial activation.^451^

Altered activation of the cannabinoid system contributes to the development of HD. Genetic deletion of CB2 receptors in the R6/2 mouse model increased behavioral impairments, shortened life duration, and accelerated microglial activation.^452^ Furthermore, stimulation of CB1 and/or CB2 receptors in microglia was demonstrated to induce M2 polarization, while knocking out CB2 receptors in mice inhibited M2 polarization.^453^ As a result, pharmacological techniques targeting CB2 receptors in microglia may be beneficial in the treatment of HD.

#### Abnormal microglia in HD

Previous studies showed that the number and morphology of microglia changed with age in both WT and R6/2 mice, with more pronounced changes observed in the transgenic mice. Structural abnormalities in microglia were observed as early as 7 weeks in R6/2 brains.^454^ The findings suggest that changes in the dynamic states of microglia may contribute to an impairment of their neuro-supportive functions in HD. It is reported that increased immunostaining for ferritin in the striatum, cortex, and hippocampus in brains of R6/2 mice and HD patients. Ferritin-labeled microglia in R6/2 mice exhibited dystrophic, and some of these cells contained mHTT. Similarly, brains from HD patients displayed elevated numbers of ferritin-containing microglia that exhibited positive staining with Perl’s stain, indicating abnormally high iron levels.^455^ These findings provide new insights into the mechanisms underlying HD pathogenesis. Microglial elimination in R6/2 mice prevented the loss of PNNs and dramatically increased PNNs in the brains of naïve littermates. These findings suggest that microglia may play a new role in regulating the integrity and formation of PNNs.^456^

## Preclinical and clinical evidence target on microglia in neurodegenerative diseases treatment

### Alzheimer’s disease

#### Regulation of neuroinflammation enhances phagocytosis of Aβ by microglia

AD patients and mouse models have elevated levels of inflammatory markers, and several AD risk genes associated with innate immune function have been identified, suggesting that neuroinflammation plays a critical role in AD pathogenesis.^457^ In AD, microglia exhibit a pro-inflammatory phenotype that impairs the phagocytic activity of microglia, promotes cerebral pathology, and exacerbates behavior defects. Therefore, some studies have improved AD by reducing microglia-mediated neuroinflammation. Min Hee Park et al. found N, N′-Diacetyl- p-phenylenediamine suppressed neuroinflammation, enhanced microglia phagocytosis, reduced Aβ burden, and improved cognitive function in AD transgenic mouse models by suppressing the expression of NLRP3 inflammasome-associated proteins.^458^ Inhibiting the NLRP3 inflammasome using NLRP3-specific inhibitor dapansutrile (OLT1177) could rescue cognitive impairment in a mouse model of AD.^459^ Besides, desloratadine, a selective antagonist of the 5HT2A receptor, and DW14006, a direct AMPKα1 activator, were applied in AD mouse models and found that they ameliorated innate immune response, enhanced microglial Aβ phagocytosis, reduced amyloid plaque deposition by polarizing microglia to an anti-inflammatory phenotype via selectively antagonizing the 5HT2A receptor and activating AMPKα1/PPARγ/CD36 signaling respectively.^460,461^ In addition, Chunmei Liang et al. found microRNA-146a lessened neuroinflammation and Aβ burden, prevented neuronal loss, and reduced cognitive deficits in APP/PS1 transgenic mice by switching microglial phenotype, decreasing pro-inflammatory cytokines, and enhancing phagocytosis.^462^ The above evidence suggests that modulating microglia-mediated neuroinflammation is a key therapeutic target in treating AD.

#### TREM2 activation modulates microglial phenotype and enhances Aβ phagocytosis

Emerging evidence has shown that TREM2 activation increased Aβ phagocytosis, relieved neuroinflammation, and improved cognitive behavior in AD mouse models. Thus, several approaches have been developed to enhance TREM2 signaling by TREM2 agonist antibody, direct delivery of the *TREM2* gene, or stimulating other pathways to increase TREM2 expression. Currently available TREM2 antibodies are as follows: (1) 4D9, with a stalk region epitope close to TREM2 cleavage site, reducing TREM2 shedding, concomitantly activating TREM2 downstream phospho-SYK signaling^171^; (2) AL002c, agonist antibody that activate TREM2 signaling,^172^ and phase I clinical trials have demonstrated that a variant of AL002c is safe and well tolerate, and a phase 2 study to evaluate efficacy and safety of AL002 in participants with early AD are in progress (NCT04592874); (3) bispecific antibody, targeting TREM2 as a tetravalent TREM2 agonistic antibody that increases TREM2 activity by 100-fold, meanwhile targeting transferrin receptor, improved antibody brain entry by more than 10-fold.^173^ These antibodies have been evaluated in AD mouse models and found that they reduced Aβ and tau pathology, tempered microglial inflammatory response, and improved cognitive behaviors.^171–173^ In addition, Pengzhen Wang et al. developed microglia-targeted gene delivery systems-coated TREM2 plasmid to upregulate the TREM2 level in the brain, significantly regulated microglial polarization toward an anti-inflammatory phenotype, relieved neuroinflammation, enhanced Aβ clearance, and improved cognitive performance in APP/PS1 mice.^463^ Qiming Xu et al. utilized cGAMP to activate the cGAMP-STING-IRF3 pathway, leading to TREM2 upregulation, which decreased Aβ burden, neuron loss, and improved cognitive impairment in AD mice.^464^ Taken together, the above evidence suggests that activation of TREM2 in microglia is beneficial in treating AD.

#### Inhibition of microglial exosome synthesis and secretion reduces pathological Tau spreading

Microglia release tau-containing exosomes that contribute to the spread of tau.^402,403^ Thus, it may be possible to halt tau propagation by suppressing microglial exosome production and secretion. The P2X purinoceptor 7 (P2RX7), an ATP-evoked Na^+^/Ca^2+^ channel, is primarily expressed in microglia and triggers exosome release. Pharmacologic blockade of P2RX7 at an early stage in tau mice significantly impaired exosome secretion from microglia, decreased tau accumulation in the brain, and improved working and contextual memory.^161^ In addition, pharmacologic inhibition of exosome synthesis by targeting neutral sphingomyelinase-2 (nSMase2) could also reduce tau secretion from microglia.^402^

#### Altering microglial metabolism to decrease amyloid burden

Microglial phagocytosis requires a great deal of energy, and metabolic dysfunction has been implicated in the pathogenesis of AD.^465,466^ Stress can cause microglia to metabolic switch from oxidative phosphorylation (OXPHOS) to aerobic glycolysis,^467^ which may facilitate the immune function of microglia. However, persistent aerobic glycolysis impaired microglial function of Aβ engulfment.^468^ Some studies have found that regulating the microglial metabolism could enhance the phagocytosis of Aβ by microglia. Rui-Yuan Pan et al. found that sodium rutin (NaR) treatment shifted microglial metabolism from anaerobic glycolysis to mitochondrial OXPHOS, providing microglia with sufficient ATP for Aβ clearance. NaR administration could attenuate synaptic impairment, neuroinflammation, and Aβ burden in brains and rescue learning and memory defects in two mouse models of AD. Moreover, NaR promoted microglial Aβ clearance also by increasing the expression levels of phagocytosis-related receptors in microglia.^469^ This finding suggests that NaR is a potential therapeutic agent for AD. Rui-Yuan Pan et al. found that H4K12la levels were increased in microglia adjacent to Aβ plaque, facilitating the switch from OXPHOS toward glycolysis in microglia by enhancing glycolytic genes expression comprising PKM2. etc. When Pkm2 was specifically ablationed in microglia, cognitive deficits in AD mice were ameliorated.^470^ Besides, Baik et al. utilized IFN-γ to boost metabolic pathways through mTOR signaling and found that AD mice exhibited decreased amyloid pathology and improved memory deficits after IFN-γ administration.^466^ These findings suggest that modulation of microglial bioenergetic pathways and metabolic state might be promising strategies to treat AD.

#### Altering the microglial phenotype to enable microglia to play a protective role

The brain microenvironment regulates microglia phenotype conversion. For example, when organotypic brain slices from old APP/PS1 mice were co-cultured with young, neonatal WT mice, old microglia cells derived from old mice moved towards the amyloid plaques and cleared the plaque halo. Conditioning media derived from young microglia can also enhance old microglia’s proliferation and phagocytosis of amyloid plaques.^471^ This evidence suggests that microglia function can be restored through a microenvironment-driven therapeutic approach. Based on this rationale, Yifei Lu et al. developed a ROS-responsive polymeric micelle system. The micelles could accumulate in the diseased regions through an Aβ transportation-mimicked pathway. These micelles normalized oxidative and inflammatory microenvironments and reeducated microglia in early AD. In vivo studies established that this system decreased Aβ plaque burdens and improved cognitive functions.^472^

In AD brains, many proteins were highly expressed by microglial cells according to proteomics analysis, and some proteins mediated a critical checkpoint in the microglia phenotype transition. For example, RIPK1 was highly expressed by microglial cells in human AD brains, and RIPK1 decreased the microglial phagocytic capacity. Pharmacological or genetic inhibition of RIPK1 reduced neuroinflammation, decreased the cerebral Aβ load, and improved the behavioral deficits by enhancing the microglial degradation of Aβ.^473^ Similarly, genetic knockdown of REV-ERBs or pharmacological inhibition of REV-ERBs with the small molecule antagonist SR8278 promoted microglia polarization toward a phagocytic phenotype and enhanced microglial engulf of Aβ. Constitutive deletion of Rev-erbalpha altered microglia phenotype and decreased amyloid plaque number and size in an AD mouse model.^474^

Photobiomodulation has been proposed as a possible therapy for AD by nonthermal exposure of tissue to low-power light at the near-infrared end of the visible spectrum to trigger biological responses in cells and tissues. Li-Huei Tsai et al. found that using noninvasive exposure to 40-Hz light for 1 h transformed microglia into an engulfing state, increased amyloid/microglia colocalization, and reduced Aβ load by 40–50% in an AD mouse model at early pre-plaque stage.^475^ Similarly, Lechan Tao et al.^476^ found that treatment with 1070-nm light pulsed at 10 Hz induced the morphological change of microglia, increased the colocalization with Aβ, reduced Aβ burden, and ameliorated cognitive and memory impairment in AD mice. The above studies suggest that controlling microglia fate towards a beneficial phenotype is feasible and worth exploring to treat AD.

### Parkinson’s disease

#### Target microglial activation and related neuroinflammation to treat PD

Microglial activation and the resulting inflammatory responses mediated are crucial factors in the development of PD. Understanding the intricacies and imbalances in microglial activation may provide insights into new therapeutic interventions for PD. Naloxone has been shown to safeguard dopaminergic neurons by impeding microglial activation and the production of pro-inflammatory cytokines.^477^ Similarly, celecoxib protects dopaminergic neurons from degeneration by suppressing microglial activation via inhibiting COX-2 in a rat model of PD.^478^ The regulation or modification of microglial receptors, such as TLRs and CB2 receptors, presents a promising pharmacological approach for the treatment of PD.^479,480^ A study in MPTP-induced PD mice found that endurance exercise was neuroprotective through modulating TLR2 and downstream signaling components, such as MyD88, TRAF6, and TAK1.^481^ The natural agonist of CB2 receptors, β-caryophyllene, and the selective CB2 receptor agonist JWH133 have both demonstrated neuroprotective effects in MPTP-induced PD mice by regulating microglial activation and suppressing expressions of pro-inflammatory cytokines.^255,482^ JWH133 also protects against BBB disruption and peripheral immune cell infiltration.^255^ These findings suggest that CB2 receptor agonists may serve as potential therapeutic targets for PD. Additionally, NLRP3 inhibitor MCC950 significantly inhibited inflammasome activation, accumulation of α-synuclein aggregates, and nigrostriatal dopaminergic degeneration, improving motor performance in PD mouse models.^223^ Several natural compounds, including ginsenoside Rg1,^483^ piperine,^484^ curcumin,^485^ rosmarinic acid,^486^ and astilbin,^487^ have been shown to inhibit microglial activation and reduce the production of pro-inflammatory cytokines in mouse models of PD. These compounds offer potential alternative therapeutic options for the treatment of PD.

#### Target microglial phenotype to treat PD

Vitamin D protects dopaminergic neurons from inflammation and oxidative stress by inhibiting microglial activation and promoting M2 polarization, with increased expression of M2 microglia markers, including CD163, CD204, and CD206.^488^ PPAR-γ agonists, such as rosiglitazone, have neuroprotective effects on dopaminergic neurons by regulating microglial polarization. Rosiglitazone reduces pro-inflammatory cytokines TNF-α and IL-1β and increases anti-inflammatory cytokines TGF-β and IL-10, induces M2 polarization, and alleviates dopaminergic neuron degeneration of SNc neurons.^489^

#### Microglia-targeted therapies in clinical trials for PD

The recruitment for the phase 1 trial of inzomelid, a promising NLRP3 inhibitor, was completed in 2020 (NCT04015076), but results have not yet been disclosed. Myeloperoxidase (MPO) is a key enzyme that produces ROS by phagocytic cells such as microglia and is a mediator of inflammatory processes in many diseases.^490^ Thus, inhibition of MPO represents an attractive target for reducing neuroinflammation in PD. AZD3241, a selective and irreversible MPO inhibitor, reduced microglial activation assessed by PET imaging in PD patients and was well tolerated.^491^ Glucagon-like peptide-1 (GLP-1) receptor agonists are commonly used to treat type 2 diabetes. GLP-1R agonist NLY01 directly prevents microglial-mediated astrocyte conversion to an A1 neurotoxic phenotype in the hA53T transgenic mice.^113^ GLP-1 analogs semaglutide and liraglutide reduced microglial activation, α-syn accumulation, the loss of dopaminergic neurons, and improved motor impairments in the chronic MPTP-induced mouse model of PD.^492^ Exenatide, one of the most extensively studied GLP-1 receptor agonists, provided both motor and cognitive benefits in PD patients and was well tolerated.^493^ Notably, the favorable effects lasted 12 months after treatment discontinuation.^494^

### Multiple system atrophy

#### Target microglial activation and related neuroinflammation to treat MSA

The effect of microglial activation inhibitors has been examined in MSA mouse models and MSA patients. Myeloperoxidase (MPO) is a key enzyme that produces ROS by phagocytic cells such as microglia and is a mediator of inflammatory processes in many diseases.^490^ MPO expression level in reactive microglia was upregulated in the brains of MSA patients and a mouse model of MSA.^495^ Thus, inhibition of MPO represents an attractive target for reducing neuroinflammation in MSA. An MPO inhibitor, when applied in the MSA mouse model in the early stage of the disease, suppressed microglial activation and neuroinflammation, reduced intracellular aggregates of α-syn, and rescued neuronal loss and improved motor deficits.^495^ In the following study, MPO inhibitor therapy was initiated in the advanced stage of the disease after the onset of severe MSA-like neuropathology. It reduced microglial activation and decreased α-syn accumulation in degenerating brain areas. However, these effects failed to improve motor impairments and rescue neuronal loss,^496^ suggesting that the timing of treatment is important, and that inhibition of microglial activation should be implemented early in the disease. Verdiperstat (MPO inhibitor) has been clinically tested in Phase 1 and Phase 2 trials on approximately 250 healthy volunteers and patients. Results from a Phase 2 trial in MSA patients with the MPO inhibitor treatment for 12 weeks showed improved clinical scores and neuroinflammation reduction measured by PET imaging (NCT02388295). Therefore, a double-blind, randomized,placebo-controlled, phase III study is planned to evaluate the efficacy and safety of Verdiperstat (BHV-3241) treatment for 48 weeks in subjects with MSA, in which UMSARS score is used to assess the efficacy of verdiperstat (NCT03952806).

However, some interventions have failed in clinical trials in patients with MSA despite showing therapeutic effects in mouse models. Minocycline belongs to second‐generation tetracyclines, which can cross the blood‐brain barrier and inhibit microglial activation and pro‐inflammatory cytokines production.^497–499^ When applied in the transgenic MSA mouse model, minocycline suppressed microglial activation and prevented loss of dopaminergic substantia nigra pars compacta neurons and striatal dopaminergic terminals.^276^ Next, a multicenter, double-blind, randomized, placebo-controlled clinical trial was performed to examine the efficiency of minocycline on 63 patients with clinically probable MSA-P (NCT00146809). However, after a 12-month treatment with minocycline, microglial activation seemed to be decreased, as demonstrated by PET imaging, but symptom severity failed to show clinical benefits, as assessed by clinical motor function assessment.^500^ Besides, Fluoxetine, a selective serotonin reuptake inhibitor, significantly reduces LPS-induced pro-inflammatory cytokines production and oxidative stress in microglia and enhances microglia phagocytosis and autophagy.^501^ Fluoxetine ameliorated motor deficits and decreased neurodegenerative pathology in MSA mice.^502^ However, a phase II double-blind, placebo-controlled study on MSA patients found no difference in progression rates (NCT01146548).^503^

### Amyotrophic lateral sclerosis and frontotemporal dementia

#### Targeting microglial neuroinflammation to treat ALS

Targeting microglial neuroinflammation has therapeutic effects in ALS mouse models. For example, the organoselenium compound diphenyl diselenide (DPDS) improved motor deficit, prolonged survival, and reduced motor neuronal loss in hSOD1^G93A^ transgenic mouse through suppressing microglia activation by inhibiting NLRP3 inflammasome activation and IΚb/NF-κB pathway.^504^ Another study also demonstrated that inhibition of NF-κB activation by daily oral administration of A Nitroalkene Benzoic Acid Derivative (BANA) could reduce reactive microglia and prolong survival in SOD1^G93A^ rats.^505^ In addition, LPS-treated mSOD1^G93A^ microglia caused microglial activation and released nitric oxide, which induced co-cultured motor neuron injury. Pretreatment with L-NIL, an inhibitor of iNOS, increased co-cultured motor neuron survival.^506^ These studies indicate that inhibiting microglial activation and related iNOS production could treat ALS.

Additionally, pathological proteins of ALS can be secreted into the extracellular matrix, and these pathological proteins are sensed by microglia, causing microglial activation and pro-inflammatory effect that are harmful to co-cultured motor neurons. For example, in primary mouse microglia, SOD1^G93A^ protein caused NLRP3 inflammasome activation and a significant increase in the release of pro-inflammatory factors such as TNF, IL-1β, superoxide, and iNOS.^507^ *Nlrp3* knockout or pretreatment with a specific NLRP3 inhibitor blocked SOD1^G93A^ protein’s ability to induce IL-1β secretion from microglia, indicating some specific pro-inflammatory factors production was NLRP3 dependent.^508^ Therefore, NLRP3 inhibition may also be used as a therapeutic strategy to prevent the progression of ALS by halting microglial neuroinflammation. These findings indicate that targeting reactive microglia and related neuroinflammation holds promise as therapeutic strategies for ALS.

#### Targeting progranulin insufficiency-caused microglial dysfunction may treat FTD-GRN

Heterozygous GRN mutations cause progranulin haploinsufficiency, resulting in patients with FTD (FTD-GRN). GRN encodes a secreted protein (progranulin) expressed in microglia and neurons in human brains. In the CNS, microglia express the highest progranulin levels and show higher expression in reactive states when responding to aging,^360^ or disease pathology.^509^ Progranulin reduction in microglia alone is sufficient to recapitulate inflammation, and lysosomal dysfunction, both of which are important pathogenic drivers in FTD-GRN.^510^ Therefore, it may be possible to treat or prevent FTD-GRN by targeting the dysfunction of microglia caused by insufficient progranulin. Telpoukhovskaia et al. screened a library of bioactive compounds using RNA-mediated oligonucleotide annealing, selection, and ligation with next-generation sequencing (RASL-seq) technique. They identified two compounds nor‑binaltorphimine dihydrochloride (nor‑BNI) and dibutyryl-cAMP, sodium salt (DB‑cAMP) that can reverse microglial defects in *Grn‑*deficient mice. Experimental evidence revealed that in progranulin‑deficient primary mouse microglia, these two compounds promoted microglial transcriptional profiles transition from the *Grn* KO signature toward the *Grn* WT state and could also partially rescue lysosomal dysfunction. As patients with FTD exhibited lysosomal dysfunction and neuroinflammation, and microglial signature is closely related to the regulation of neuroinflammation, thus this study proposed two compounds that may be beneficial for FTD therapy, meanwhile also highlighting the potential of a transcription‑based platform for drug screening.^510^

### Progressive supranuclear palsy

#### Inhibition of microglia activation as a therapeutic target to treat PSP

5-lipoxygenase (5-LO), broadly expressed in the CNS, is a potent pro-inflammatory mediator regulating microglial activation.^511^ 5-LO levels were significantly higher in the frontal cortex of the brains of PSP patients than in controls.^512^ By employing an AAV vector system to over-express 5-LO in the brain of the same transgenic model of human tauopathy, 5-LO increased tau phosphorylation and glial activation and enhanced behavioral impairments.^513,514^ In contrast, genetic deletion of 5-LO in P301S tau mice^515^ or pharmacologic inhibition of 5-LO with zileuton,^512,516,517^ both resulted in significant reduction of tau phosphorylation and glial cell activation and thus ameliorated cognitive deficits. It is worth mentioning that administration of 5-LO blocker at both early (age of 3 months)^516^ and late (age of 12 months)^517^ stages showed a beneficial effect in the transgenic tau mice, which suggests that the inhibition of 5-LO has a protective effect throughout the disease course. Collectively, the above evidence provides a rationale that 5-LO is a viable therapeutic target for treating and even preventing human tauopathy.

Benfotiamine (BFT) is a synthetic vitamin B1 (thiamine) derivative that reduces inflammation in LPS-stimulated BV-2 microglia.^518^ One previous study found that BFT reduced phosphorylated tau levels, ameliorated Aβ load, and improved cognitive function in an AD mouse model APP/PS1 mice.^519^ Another study treated P301S tau transgenic mice with chronically BMT dietary from the age of 1 to 10 months and found that BFT significantly decreased the expression of inflammatory mediators, including COX-2, TNF-α, IL-1β, NF-κB p65, and iNOS, attenuated oxidative damage, as well as ameliorated mitochondrial dysfunction and motor neuron loss, resulting in behavior improvement and lifespan increased.^520^ These findings demonstrate that BFT is a promising therapeutic agent for treating tau pathologies such as AD, PSP, and FTD.

### Huntington’s disease

#### Inhibition of microglial activation as a therapeutic target to treat HD

There is no disease-modifying therapy for HD, so current treatment focuses on chorea and mental symptoms. Several medicines, including immunomodulators, and cytokine-neutralizing antibodies, have been explored in various research in the hunt for successful therapy. The expression of the CB2 cannabinoid receptor was increased in striatal microglia of HD patients and transgenic mouse models. Genetic ablation of CB2 receptors in R6/2 mice enhanced microglial activation, aggravated disease symptoms, and reduced lifespan. In contrast, activation of CB2 receptors with selective agonists reduced neuroinflammation, striatal neuronal loss, and motor symptoms in WT mice subjected to excitotoxicity.^452^ The selective CB2 receptor agonist, SR144528, could protect the striatum against mitochondrial toxin malonate toxicity via their effects on glial cells, especially reactive microglia.^521^ The above evidence suggests that CB2 receptors could be a potential therapeutic target for slowing neurodegeneration in HD. Galectin-3 (Gal3) is a lectin that was upregulated in the plasma and brain of patients and mice with HD, and their plasma Gal3 levels correlated with disease severity. Its upregulation in microglia occurred prior to the onset of motor impairment and contributed to inflammation through NF-κB- and NLRP3 inflammasome-dependent pathways. Knockdown of Gal3 suppressed neuroinflammation, reduced the aggregation of mHTT, alleviated motor dysfunction, and increased survival in HD mice.^522^ These findings suggest that Gal3 could be a novel target for therapeutic intervention in HD. Poly (ADP-ribose) polymerase-1 (PARP-1) is an enzyme involved in both DNA repair and transcriptional regulation. Inhibiting PARP-1 suppresses microglial activation likely via regulating NF-kB-dependent gene transcription.^523^ In a traumatic brain injury mouse model, administration of a PARP inhibitor for 12 days reduced inflammation, increased neuronal survival, and improved performance.^524^ Olaparib, a PARP-1 inhibitor commercially available as an anti-neoplastic drug, was found to have a neuroprotective effect in the R6/2 mouse model of HD. Administering Olaparib from the pre-symptomatic stage increased survival and improved clinical outcomes mainly by modulating the inflammasome activation.^525^ These results suggest that Olaparib could be a potentially useful therapy for HD. The researchers also discovered a compound (MIND4–17) that can selectively activate nuclear factor-erythroid 2 p45-derived factor 2 (NRF2) signaling by modifying a critical stress-sensor cysteine (C151) of an E3 ligase substrate adaptor protein Kelch-like ECH-associated protein 1 (KEAP1). Interestingly, the genetic correction of the disease-causing mutation restored a muted NRF2 activation response in HD neural stem cells. Conversely, selective activation of NRF2 signaling decreased the release of pro-inflammatory cytokines in primary microglia from HD mice.^526^ Semaphorin 4D (SEMA4D) is a transmembrane signaling molecule that modulates various processes central to neuroinflammation and neurodegeneration. Blocking SEMA4D significantly inhibits microglial activation and neuroinflammation in experimental autoimmune encephalomyelitis.^527^ In the YAC128 transgenic HD mouse model, treatment with an anti-SEMA4D monoclonal antibody reduced brain atrophy, improved cognition, and reduced anxiety-like behavior.^528^ These findings suggest that targeting microglia could be a promising direction in exploring treatments for HD.

**Fig. 6:**
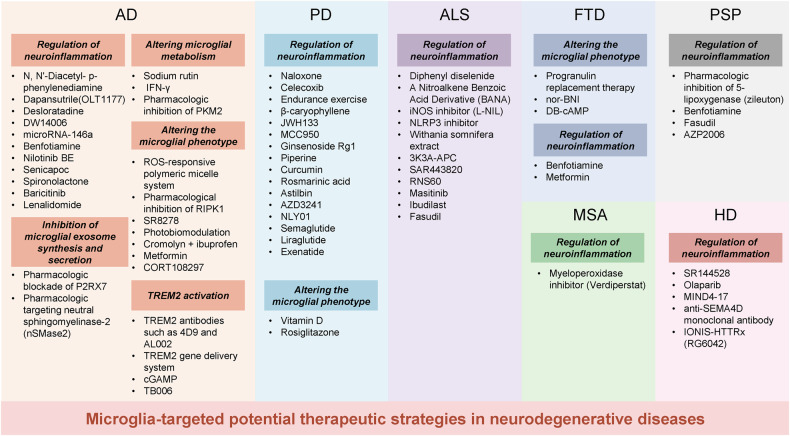
Possible microglia-targeted interventions and treatments against neurodegenerative diseases. Proof-of-principle therapeutic strategies used in cell experiments, animal studies, and clinical trials are shown together. Regulation of neuroinflammation, inhibition of microglial exosome synthesis and secretion, altering microglial metabolism, altering the microglial phenotype, and TREM2 activation are potential therapeutic strategies in treating neurodegenerative diseases. Among these, modulating neuroinflammation is the most widely used therapeutic target

Microglial activation in HD is caused by neuronal mHTT-mediated excitotoxicity or mHTT expression in microglia.^529^ Several therapeutic approaches are being investigated to reduce the expression of mutant huntingtin RNA (Htt RNA), which could potentially attenuate microglial activation, alleviate inflammatory processes, and provide neuroprotection. Two selective ASOs, WVE-120101 and WVE-120102, which target the most often occurring SNPs in HD patients, have been investigated in human studies (NCT04617847, NCT04617860). However, results showed no significant change in mHTT protein in CSF. The HTT-targeting ASO IONIS-HTTRx (also known as ISIS 443139 and RG6042), which is thought to act through the RNase H1 mechanism, has completed a Phase 1/2a clinical trial (NCT02519036, NCT03342053) and is currently undergoing a large multicenter worldwide efficacy study. This therapy has been deemed safe, with no significant side effects reported in the treatment group. Furthermore, dose-dependent reductions of mHTT concentrations in CSF have been observed, with a 40% decrease in mHTT at the two highest medication dosages,^530^ which holds promise for disease-modifying treatments for HD. Preclinical evidence in mouse models and clinical trials in patients with neurodegenerative disease has been provided in Table 1. The list of potential microglia-targeted interventions and treatments against neurodegenerative diseases is provided in Fig. 6.

**Table 1:** Preclinical evidence in mouse models and clinical trials in patients with neurodegenerative disease

Intervention	Mechanism	Preclinical evidence			Clinical Trial			
		Mouse models	Treatment duration	Main findings	Design	Sample size	Treatment duration	Main findings or progress
Alzheimer’s disease (AD)								
AL002	Agonist antibody that activates TREM2 signaling to promote microglial clearance of Aβ	5×FAD mice expressing either the common variant or the R47H variant of TREM2	Initiated weekly i.p. injections of AL002c or a control IgG at 5 mo of age, and continued until 8 mo of age	Tempered microglial inflammatory response, increased microglial phagocytosis of Aβ, and improved behavior^172^	Multicenter, randomized, double-blind, placebo-controlled	265 early AD	48–96 weeks	Phase 2 status: recruiting starte d at 22 Jan 2021 and estimated end at Jan. 2024 (NCT04592874)
Cromolyn + ibuprofen	Modulate microglia to promote clearance of Aβ	APPSwedish-expressing Tg2576 mice	Initiated i.p 3 times per week at 5 mo of age, and continued for 3 mo	Cromolyn, alone, or in combination with ibuprofen, increased microglial recruitment to, and phagocytosis of Aβ^531^	Multicenter, randomized, double-blinded, placebo-controlled	620 early AD	72 weeks	Phase 3 status: completed n o results posted. (NCT02547818)
Nilotinib BE	Tyrosine kinase inhibitor	C57BL6 mice	A 7-day course of daily i.p.injections with nilotinib before i.p.injection with LPS	Modulated neuroinflammatory responses of microglia and astrocyte and improved cognitive function.^532^	Multicenter, randomized, double blind, placebo-controlled	1275 Early AD	72 weeks.	Phase 3 Status: not yet recruiting estimated started at 1 Feb 2022 and estimated end at 31 Dec 2025 (NCT05143528)
TB006	Monoclonal antibody targeting galectin 3, stimulate TREM2-DAP12 signaling to regulate microglia activation	Two AD transgenic mice (APPSwe, 5×FAD) and Aβ injection induced mice	Two-week treatment with mTB001, a surrogate of TB006	Reduced aggregation of Aβ/Tau proteins and neuroinflammation, and significant improvement of cognitive performance (https://www.truebinding.com/)	Multicenter open-label long-term extension study	180 de novo AD	113 weeks (101 weeks of dosing and a 12-week safety follow-up period)	Phase 2 Status: Active, not recruiting Started at 14 Sep 2022 and estimated end at Oct. 2024 (NCT05476783) A Phase 1/2 study concluded that TB006 imporved cognitive function and reduced Aβ load. (NCT05074498, *n* = 140)
Senicapoc	Ca^2+^-activated potassium channel KCa3.1 inhibitor	5×FAD mice	Initiated treatment at 6 mo of age, and continued for 3 mo	Reduced microglia-mediated neuroinflammation, decreased Aβ load, and enhanced hippocampal neuronal plasticity.^533^	Randomized double-blind, placebo-controlled	55 MCI and mild AD	52 weeks	Phase 2 status: recruiting st arted at 18 Mar 2022, and estimated end at Jun 2025 (NCT04804241)
Spironolactone	Inhibit microglial release of proinflammatory cytokines	Sprague-Dawley rats with Aβ injected in the dorsal hippocampus for four consecutive days	I.p. injection spironolactone or vehicle for 14 days.	Significantly lowered Iba1 protein levels^534^	Randomize, double-blind, placebo-controlled	30 MCI and early AD	12 mo	Phase 4 status: recruiting start ed at 6 Sep 2022 and estimated end at Sep 2023 (NCT04522739)
Metformin	Modulation of microglia phenotype	C57BL6/J mice	Initiated at 18 mo and treatment for 10 weeks	Promoted microglia conversion into an anti-inflammation phenotype, improved cognitive function^535^	Randomized, double blinded, placebo controlled	370 MCI	24 mo	Phase 2/3 status: recruiting started at 22 Mar 2021, and estimated end at 30 Apr 2026 (NCT04098666)
CORT108297	A selective glucocorticoid receptors antagonist; changed the phenotype of microglia from an activated to a quiescent form.^536^	Sprague-Dawley rats	1 week after icv injection of Aβ, two times i.p. injections per day for 1 week	Reduced microglia-mediated neuroinflammation and improved cognitive function.^537^	Randomized, double-blind, crossover assignment	52 MCI due to AD	2 weeks	Phase 2 status: recruiting started at 28 Jun 2021 and estimated end at 1 Jan 2024 (NCT04601038)
Lenalidomide	A thalidomide derivative inhibiting microglia activation and the production of pro-inflammatory cytokines	An animal model of PD (mThy1-α-syn transgenic mice)	Initiated at 9 mo and treatment for 5 weeks	Reduced microglial activation and behavioral deficits^538^	Randomized, double-blind, placebo controlled	30 MCI due to AD	Daily orally treatment for 12 mo followed by 6 mo washout	Phase 2 status: recruiting started at 22 Jul 2020 and estimated end at Sep 2024 (NCT04032626)
Parkinson’s disease (PD)								
MCC950/Inzomelid	NLRP3 inhibitor, significantly inhibited microglial inflammasome activation	6-OHDA model of PD + PFF model of PD	At 21 days after 6-OHDA lesioning α-synuclein PFF-injected mice at 30 days^223^	Abolished α-synuclein-mediated NLRP3 activation and ASC release in microglia^223^	Single-center, double blind, randomized, cross-over	80 healthy adult participants	Up to 16 days	Phase 1 completed re sults pending. (NCT04015076).
AZD3241 (MPO inhibitor)	Enzyme myeloperoxidase inhibition (myeloperoxidase is a reactive oxygen generating enzyme and is expressed by microglia.)	MPTP model	[AstraZeneca data on file]	Suppression of microglia activity and neuroprotective effect on dopamine cell survival [AstraZeneca data on file]^491^	Multicentre, double-blind, randomized, placebo-controlled, parallel-group	51 PD	12 weeks	Phase 2 (NCT01603069);
	–	–	–	–	Multi center, double-blind, randomized, placebo-controlled, parallel-group	24 PD	8 weeks	Phase 2, Microglial activation measured by PET imaging. (NCT01527695)
GLP-1 receptor agonists, NLY01	GLP-1R activation	α-synuclein (hA53T) transgenic mouse model	Started at six mo of age (for five mon)	Prevent microglial-mediated conversion of astrocytes to an A1 neurotoxic phenotype^113^	Multicenter, randomized, double-blind, placebo-controlled	255 early untreated PD	36 weeks	Phase 2 (NCT04154072)
GLP-1 receptor agonists, Exenatide	–	–	–	–	Multicentre, double-blind, randomized, placebo-controlled	200 mild to moderate PD	96 weeeks	Phase 3 Started at Jau 20, 2020 and estimated end at Jun 30, 2024 (NCT04232969)
Multiple system atrophy (MSA)								
Minocycline	Belongs to tetracyclines. Cross BBB. Inhibits microglial activation and neuroinflammation	PLP-α-syn mouse	Treatment for 2 mo starting at 2 mo of age before motor symptom onset	Suppressed microglia activation, prevented neuronal loss^276^	Multicenter, randomized, double-blind, placebo-controlled	63 probable MSA-P	12 mo	Phase 3 status: completed reduc tion of microglia activation assessed by PET, but symptom severity unchanged by clinical assessment^500^ (NCT00146809)
Verdiperstat (MPO inhibitor)	MPO is a key enzyme involved in the production of ROS in microglia and is a mediator of inflammatory processes	PLP-α-syn mouse +3NP model	Treatment for 28 days starting at early stage (6 mo of age)	Suppressed microglia activation, reduced a-syn accumulation, rescued neuronal loss and improved motor deficits^495^	Multicenter, randomized, double-blind, placebo- controlled	59 probable or possible MSA	12 weeks of verdiperstat (AZD3241)	Phase 2 status: completed improvemen t on clinical scores and reduced neuroinflammation measured by PET imaging (NCT02388295)
	–	PLP-α-syn mouse +3NP model	Treatment for 20 days starting at advanced stage (8-9 mo of age)	Reduced microglia activation and a-syn accumulation, but motor impairments and neuronal loss were unchanged^496^	Multicenter, randomized, double-blind, placebo-controlled	336 probable or possible MSA	48 weeks of verdiperstat (BHV-3241)	Phase 3 status: active, not recruiting UMSARS score is used to assess the clinical efficacy (NCT03952806)
Fluoxetine	SSRI, enhances microglia phagocytosis, reduces microglia inflammation	MBP-α-syn mouse	Treatment for 28 days starting at motor symptom onset (6 mo of age)	Ameliorated motor deficits, decreased neurodegenerative pathology, increased GDNF and BDNF^502^	Multicenter, randomized, double-blind, placebo-controlled	87 MSA	6 mo	Phase 2 status: completed n o change in rate of progression^503^ (NCT01146548)
Progressive Supranuclear Palsy (PSP)								
AZP2006	Increased levels of progranulin, inhibited microglial activation and proinflammatory cytokine production, and decreased tau phosphorylation	SAMP8 (Senescence Accelerated Mouse Prone-8) mouse model	Administrated at 2 mo, 4 mo, and 6 mo of age, respectively	Inhibits microglial activation and related neuroinflammation, prevent reversed cognitive defects.^539^	Multicenter, randomized, double-blind, placebo-controlled	36 PSP	84 days.	Phase 2 status: active, not recruiting star ted at 22 Jun 2020 and estimated end at 16 Jul 2022 (NCT04008355)
Fasudil	Rho kinase inhibitor, inhibited microglial activation and promoted their transformation to an anti-inflammatory phenotype^540^	APP/PS1 Tg mice	Initiated daily i.p. injections of Fasudil at 8 mo of age, and treatment for 2 mo	Inhibits the activation of microglial and astrocytes, and improved the cognitive deficits^541^	Open label, single arm assignment	15 PSP and CBD	48 weeks	Phase 2 status: active, not recruiting sta rted at 22 Jan 2021 and estimated end at 30 Nov 2023 (NCT04734379)
Amyotrophic lateral sclerosis (ALS)								
Fasudil	Rho kinase inhibitor, inhibited microglial activation and promoted their transformation to an anti-inflammatory phenotype^540^	SOD1^G93A^mice	Administrated in drinking water from 5 weeks to dead	Reduced motor neuron loss, slowed disease progression, and increased survival time^542^	Multicenter, randomized, double-blind, parallel controlled	120 ALS	20 days	Phase 2 status: active, not recruiting st arted at 20 Feb 2019 and estimated end date at Jul 2023 (NCT03792490)
Ibudilast	Phosphodiesterase inhibitor, suppresses proinflammatory microglial activation^543^	–	–	–	Multicenter, randomized, double-blind, placebo-controlled study followed by an open-label extension phase	230 ALS	Double-blind phase (12 mo) + Open-label extension phase (six mo)	Phase 2/3 status: recruiting st arted at 28 May 2020, and estimated end at Dec 2024 (NCT04057898)
Masitinib	Tyrosine kinase inhibitor, reduces microglial activation	SOD1^G93A^ rats	Initiated 7 days after paralysis onset	Decreased microgliosis, and motor neuron pathology in the degenerating spinal cord, prolonged post-paralysis survival by 40 %^544^	Multicenter, randomized, double-blind, placebo-controlled	495 ALS	Add-on therapy to riluzole for 48 weeks	Phase 3 status: recruiting st arted at 2 Feb 2021 and estimated end at Dec 2023 (NCT03127267) Phase 2/3 *n* = 394 ALS, Results show that masitinib add-on therapy to riluzole for 48 weeks at 4.5 mg/kg/d can benefit patients with ALS.^545^
RNS60	Suppresses microglial NF‐κB^546^	SOD1^G93A^ mice	I.p. every other day starting at the disease onset	Activated phagocytic microglia and increased anti-inflammatory molecules, slowed the disease progression^547^	Multicenter, randomized, double-blind, placebo-controlled	147 ALS	Add-on therapy to riluzole for 24 weeks administered by intravenous infusion once/week and inhaled via nebulization every morning	Phase 2 status: not yet recruiting estim ated start at Nov 2022 and end at Jun 2024 (NCT02988297) Phase 2 The mean rate of decline in respiratory function, the eating and drinking domain ability was slower in the RNS60 arm.^548^ (NCT03456882)
Minocycline	Suppresses proinflammatory microglial activation	SOD1^G37R^ mice	Initiated at the age of 7 or 9 mo and continued treatment until the the mice reached end-stage disease	Inhibited microglial activation, delayed the onset of motor neuron degeneration, declined muscle strength, and increased the longevity of mice^549^	Multicenter, randomized, double-blind, placebo-controlled	412 ALS	9 mo	Phase 3 status: completed min ocycline has a harmful effect on patients with ALS^550^ (NCT00047723)
SAR443820	Receptor-interacting protein kinase inhibitor, inhibit inflammatory microglia	SOD1^G93A^mice	Initiated at 2.5 mo of age for 4 weeks	Inhibited inflammatory microglia.^551^	Randomized, double-blind, placebo-controlled	261 ALS	24-week RCT followed by open label up to week 106	Phase 2 status: recruiting s tarted at 13 Apr 2022, and estimated at 12 Aug 2025 (NCT05237284)
3K3A-APC	Prevents microglial activation, inhibits NLRP3 inflammasome^552^	ALS mouse model	–	Slowed disease progression and extended survival (https://www.scripps.edu/news)	Non-Randomized, open label	16 ALS	45 days	Phase 2 status: completed no re sults posted (NCT05039268)
Withania somnifera extract	Blocks NF-kB transcription and inhibit microglial activation^553^	SOD1^G93A^ mice	Fed in saline beginning at 50 days of age and continued till the mice were capable to ingest	Reduced glial activation, increased longevity, improved motor performance, and increased number of motor neurons in lumbar spinal cord.^554^	Randomize, double-blind, placebo-controlled	75 ALS	8 weeks	Phase 2 status: recruiting sta rted at 19 Oct 2021, and estimated end at Sep 2022 (NCT05031351)
Verdiperstat	See Verdiperstat in MSA section	–	–	–	Multicenter, randomized, double blind, placebo-controlled	167 ALS	24 weeks	Phase 2/3 status: active, not recruiting star ted at 28 Jul 2020, and estimated end at Apr 2023 (NCT04436510)
Frontotemporal Dementia (FTD)								
Metformin	Modulation of microglia, proinflammatory cytokines, and autophagy, decreases expression of toxic proteins produced from the C9orf72 repeat expansion	C57BL6/J mice	Initiated at 18 mo and treatment for 10 weeks	Promoted microglia into an anti-inflammation phenotype, improved cognitive function^535^	Open label, single group assignment	58 C9-ALS/FTD	24 weeks	Phase 2 status: recruiting st arted at 10 Jan 2020, and estimated end at 6 Apr 2023 (NCT04220021)
Huntington’s Disease (HD)								
VX15 (pepinemab)	A Humanized IgG4 anti-SEMA4D antibody; Inhibits microglial activation and neuroinflammati-on	YAC128 transgenic HD mouse model	Initiated at 6 weeks of age, and until they reached 12 months of age.	Reduced brain atrophy, improved cognition, and reduced anxiety-like behavior^528^	Randomized, double-blind, placebo-controlled	301 late prodromal and early manifest HD	18 mo	Phase 2 status: completed no results posted (NCT02481674)
IONIS-HTTRx also known as ISIS 443139 and RG6042	Non-selective ASO binding to HTT RNA and activating RNase H-mediated degradation	Transgenic BACHD mice containing a full-length human mutant *HTT* gene	Two weeks’ continuous intraventricular infusion	Reduce the production of toxic mRNA and RAN proteins, reducing microglia activation^555^	Randomized, double-blind, placebo-controlled	46 early HD	Every 4 weeks for four doses	The therapy was deemed safe, with no significant side effects. (NCT02519036).
	–	–	–	–	An Open-Label Extension Study	46 early HD	Every 28 days intrathecally for 14 months.	Phase 2 (NCT03342053)
WVE-120101	ASO targeting the most often occurring SNPs in HD	–	–	–	Multicenter, randomized, double-blind, placebo-controlled	61 early HD who carry a targeted SNP rs362307 (SNP1)	210 days	Phase 1b/2a (NCT03225833)
	–	–	–	–	Multicenter, open-label extension Study	27 HD who carry a SNP1	Maximum of 45 weeks of treatment	No significant change in mHTT protein in HD (NCT04617847)
WVE-120102	–	–	–	–	Multicenter, randomized, double-blind, placebo-controlled	88 early HD who carry a targeted SNP rs362331(SNP2)	210 days	Phase 1b/2a (NCT03225846).
	–	–	–	–	Multicenter, open-label extension study	36 HD who carry a SNP2	Maximum of 12 monthly doses	No significant change in mHTT protein in HD patients (NCT04617860)

*mo* month, *i.p.* intraperitoneal, *icv* intracerebroventricular, *TREM2* Triggering receptor expressed on myeloid cells 2, *MPO* Myeloperoxidase, *ROS* reactive oxygen species, *SSRI* Selective serotonin reuptake inhibitor, *MBP* myelic basic protein, *PLP* proteolipid protein, *AD* Alzheimer’s disease; *PD* Parkinson’s disease, *MSA* Multiple system atrophy; *PSP* Progressive Supranuclear Palsy, *CBD* Corticobasal Degeneration, *FTD* Frontotemporal Dementia, *HD* Huntington’s Disease, *C9-ALS/FTD* ALS and FTD patients with expansions of a hexanucleotide repeat (GGGGCC) in the C9orf72 gene, *ASO* antisense oligonucleotide

## Conclusion

Understanding of microglial roles in neurodegenerative diseases has increased exponentially in recent years. With the development of scRNA-seq and snRNAseq, microglial gene expression signatures are being defined at the single-cell level. It is demonstrated that some identified microglia in specific states correlated with pathological hallmarks and were associated with specific functions. Microglia participate in the progress of neurodegenerative diseases through multiple mechanisms. Dysregulation of microglia may cause impaired phagocytosis of pathological deposits, pathological deposits propagation, neuroinflammation, and microglial phenotype switching, and thus lead to pathology progression and neurodegeneration. Peripheral immune cells infiltration shapes microglia into a pro-inflammatory phenotype and accelerates disease progression. Dysfunctional microglia may also promote the clearance of synapses and perineuronal nets and impair neuronal plasticity and activity. Notably, microglia are a double-edged sword. Microglia can limit the propagation of Aβ and tau by phagocytosis of these proteins, but it can also contribute to neurodegeneration by accelerating their spreading. Future research will focus on precisely regulating microglia and promoting their conversion into a protective phenotype.

Recently, GWAS have demonstrated that most AD risk genes were highly or exclusively in microglia, suggesting that microglia play an important role in the development of AD. Thus, numerous studies have applied microglia-targeted therapeutic strategies in cell experiments, animal studies, and clinical trials in AD and other neurodegenerative diseases. Regulation of neuroinflammation, inhibition of microglial exosome synthesis and secretion, altering microglial metabolism, altering the microglial phenotype, and TREM2 activation are potential therapeutic strategies in treating neurodegenerative diseases. Among these, modulating neuroinflammation is the most widely used therapeutic strategy.

In spite of significant advances in the understanding of microglial functions in neurodegenerative diseases in recent years, microglial heterogeneity and dynamics during the progression of disease still need to be better clarified. The recent advent of high throughput omic data analyses help identify and determine the functional roles of microglia that are in specific states. Additionally, the advancement facilitated the discovery of dysregulated pathways and key molecules critical to pathogenesis, which will contribute to the development of new therapeutic strategies. In the next decade, research on microglia in neurodegenerative diseases should include (i) identify different microglia clusters and understand their functions (ii) identify regulatory factors of specific microglia phenotype and re-educate microglia into a protective state in the early phase of disease progress (iii) reveal microglial crosstalk with other cell types; and (iv) generate chimeric mice using xenotransplantation to recapitulate human microglial biology in vivo; (v) obtain primary microglia from fresh postmortem brain tissues of patients with various neurodegenerative diseases or reprogram human stem cells to develop into microglia-like cells to expand the studies from mouse models to human patients. These steps are essential for any successful effort to develop microglia-targeted therapeutic strategies for treating neurodegenerative disease.
